# Molecular dissection of the *Cereibacter sphaeroides* spheroidene monooxygenase

**DOI:** 10.1042/BCJ20260276

**Published:** 2026-08-13

**Authors:** Elizabeth C. Martin, Karolina O. Panas, Felix S. Morey-Burrows, Jack H. Salisbury, Joshua R. Barrett, George A. Sutherland, C. Neil Hunter, Andrew Hitchcock

**Affiliations:** 1 Plants, Photosynthesis and Soil, School of Biosciences, University of Sheffield, Sheffield, U.K.; 2 Molecular Microbiology: Biochemistry to Disease, School of Biosciences, University of Sheffield, Sheffield, U.K.

**Keywords:** carotenoids, CrtA, purple bacteria, Rhodobacter, spheroidene, spheroidenone

## Abstract

In some purple phototrophic bacteria, the enzyme spheroidene monooxygenase (CrtA) catalyses the final step of carotenoid biosynthesis, introducing a keto group at the C2 position of spheroidene to produce spheroidenone. CrtA from the model purple bacterium *Cereibacter* (previously *Rhodobacter*) *sphaeroides* has a C-terminal extension consisting of a disordered, proline-rich sequence followed by a short region containing a significant proportion of glycine residues; the role of this extension is not understood. In addition to the accumulation of spheroidene, a *C. sphaeroides* Δ*crtA* mutant generated in a previous study had a slower growth rate and made fewer photosynthetic complexes than the wild-type strain. We show here that these phenotypes are largely due to a polar effect of the *crtA* deletion on the downstream *bchID* bacteriochlorophyll biosynthesis genes. We generated a *crtA* mutant where *bchID* expression was not interrupted and used this background to test a series of CrtA C-terminal truncations to identify the minimal enzyme that retains spheroidene monooxygenase activity. Using structural modelling, we identify a histidine residue that acts as the axial ligand to a heme group required for spheroidene monooxygenase activity, and two threonine residues found proximal to the bound carotenoid. We show that the subtle photoheterotrophic growth phenotype of this new Δ*crtA* mutant is due to the loss of the C-terminus of the enzyme rather than the altered carotenoid content of this strain. We further demonstrate that the C-terminal extension appears to mediate the association of CrtA with other membrane proteins, forming a range of high-molecular-mass complexes.

## Introduction

Carotenoids are natural pigments produced by an array of microorganisms and plants, and they are responsible for much of the colouration in the natural world. In photosynthesis, carotenoids act as accessory light-harvesting pigments, increasing the usable portion of the solar spectrum by absorbing light primarily in the blue (450–550 nm) region and transferring this energy to (bacterio)chlorophyll ((B)Chl) molecules [1]. Carotenoids also play important roles in photoprotection and act as structural components of light-harvesting and reaction centre complexes [2,3,4,5].

**Figure 1:**
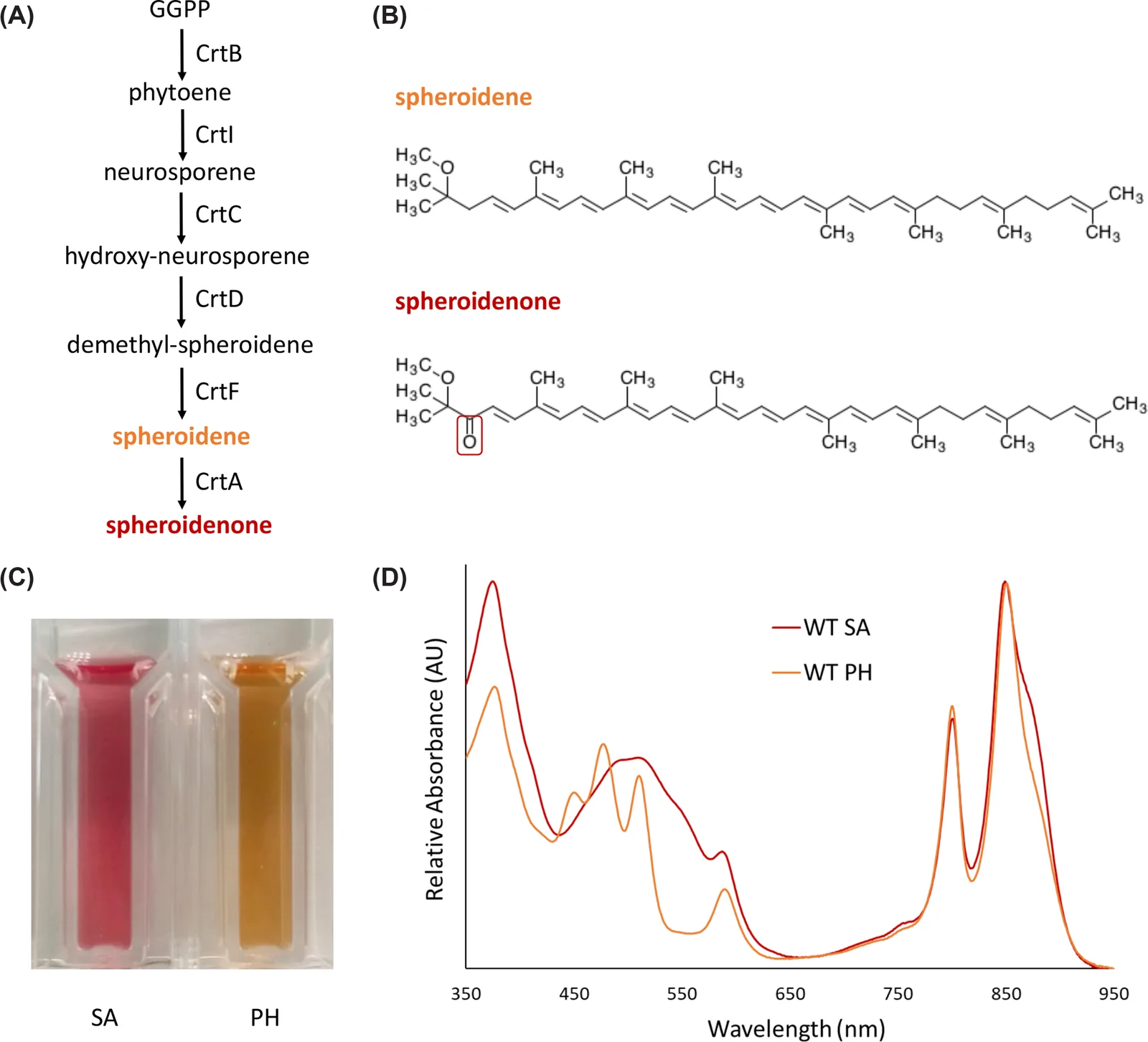
The role of CrtA in the carotenoid biosynthesis pathway (**A**) The carotenoid biosynthesis pathway of *C. sphaeroides* from the precursor geranylgeranyl pyrophosphate (GGPP). (**B**) Molecular structures of spheroidene and spheroidenone, with the keto group at the C2 position of the latter highlighted by the red box. (**C**) Photograph showing the colour of isolated ICMs from wild-type (WT) *C. sphaeroides* cells grown under semi-aerobic (SA) or photoheterotrophic (PH) conditions. (**D**) Absorption spectra of the membranes in panel (C) normalised at 850 nm highlighting the difference in the ∼450–575 nm region of the spectra due to the altered carotenoid content.

Purple phototrophic bacteria are anoxygenic phototrophs that use BChls and carotenoids for photosynthesis. In the model species *Cereibacter sphaeroides* (previously *Rhodobacter sphaeroides*), these pigments are incorporated into two light-harvesting complexes within specialised intracytoplasmic membrane (ICM) vesicles [6,7]: the peripheral light-harvesting complex 2 (LH2) and the reaction centre light-harvesting complex 1 (RC–LH1) core complex. LH2 is a nonameric ring composed of pairs of αβ subunits, where each pair binds three BChl molecules and one carotenoid [8]. RCs are surrounded by a ring of 14 pairs of LH1 α and β subunits, where each pair binds two BChls and two carotenoids, and RC-LH1 can form monomeric or dimeric structures [9–13]. The carotenoids produced by *C. sphaeroides* are dependent on the growth conditions: under strict anaerobiosis, spheroidene is produced; however, in the presence of oxygen, spheroidene is converted to spheroidenone [14]. This reaction is catalysed by spheroidene monooxygenase (CrtA/Rsp_0272; Uniprot accession: Q3J189) (Figure 1A), an enzyme unique to the purple non-sulphur bacteria (PNSB) that introduces a keto group at the C2 position of spheroidene (Figure 1B) [15,16]. CrtA also adds keto groups to both the C2 and C2′ positions of spirilloxanthin, yielding 2,2′-diketo-spirilloxanthin [17], although organisms that exclusively use the spirilloxanthin pathway lack this enzyme [18].

When grown in semi-aerobic conditions, production of spheroidenone gives *Cereibacter* and *Rhodobacter* species their distinctive red colour, whereas, in the absence of oxygen, spheroidene accumulation makes the cells orangey-yellow (Figure 1C). The absorption spectra of purified ICMs of wild-type (WT) *C. sphaeroides* cells grown in either semi-aerobic (microoxic) or photoheterotrophic (anaerobic) conditions show the distinct difference in the 450–575 nm region due to the presence of the different carotenoids (Figure 1D). Changing the carotenoids from spheroidene to spheroidenone has functional significance, by providing photoprotection under conditions where both light and oxygen are present. Ultrafast transient absorption spectroscopy measurements on RC–LH1 complexes of *C. sphaeroides* containing either spheroidene or spheroidenone showed that the extended conjugated double-bond system of spheroidenone decreases the energy of its lowest triplet state so that it can act as an effective quencher of singlet oxygen [19]. Photoprotection against singlet oxygen does not come at the cost of light harvesting, however, because the incorporation of spheroidenone into LH1 also activates an intramolecular charge-transfer channel for energy transfer to the B875 BChls [19].

A *C. sphaeroides* Δ*crtA* mutant accumulates spheroidene even in the presence of oxygen, resulting in a consistent carotenoid content that is independent of the oxygen tension within the growth medium [20], and deletion of *crtA* from the betaproteobacterium *Rubrivivax* (*Rvi*.) *gelatinosus* also abolished keto-carotenoid accumulation [21]. Previous deletion of *crtA* from *C. sphaeroides* also increased the RC–LH1 dimer:monomer ratio and resulted in a significant reduction in the levels of RC–LH1 and LH2 complexes, leading to a reduced rate of photoheterotrophic growth [20,22]. Although the basis of these phenotypes has not been elucidated, we predicted it is largely due to disrupted transcription of downstream BChl biosynthesis genes. Here, we confirmed this hypothesis by generating a new Δ*crtA* strain free from polar effects on *bchID*, allowing us to specifically study the functions of CrtA.

Purified *Rhodobacter* (*Rba*.) *capsulatus* and *Rvi. gelatinosus* CrtA enzymes require molecular oxygen and reduced ferredoxin to catalyse a proposed monooxygenase reaction, where consecutive hydroxylation reactions and the loss of a water at each step result in the formation of the C2 keto group [15]. CrtA appears to bind a penta-coordinated heme but unlike other monooxygenases it is not a classical P450 enzyme as the heme does not bind carbon monoxide in the reduced form [23], and the heme iron appears to be ligated via a histidine rather than the cysteine characteristic of P450s. Another notable feature of CrtA from *C. sphaeroides* is the extended C-terminus, which is predicted to be largely disordered, but its functional significance has not been explored. Using our new Δ*crtA* strain the aims of the present study were three-fold: (1) to dissect the CrtA enzyme to determine the enzyme core responsible for spheroidenone production; (2) to confirm the importance of the bound heme cofactor for spheroidene monooxygenase activity and investigate other residues that may constitute the active site; and (3) to elucidate the role(s) of the disordered C-terminal extension.

## Results

### Generation of a *crtA* null mutant that does not affect production of photosynthetic complexes

**Figure 2:**
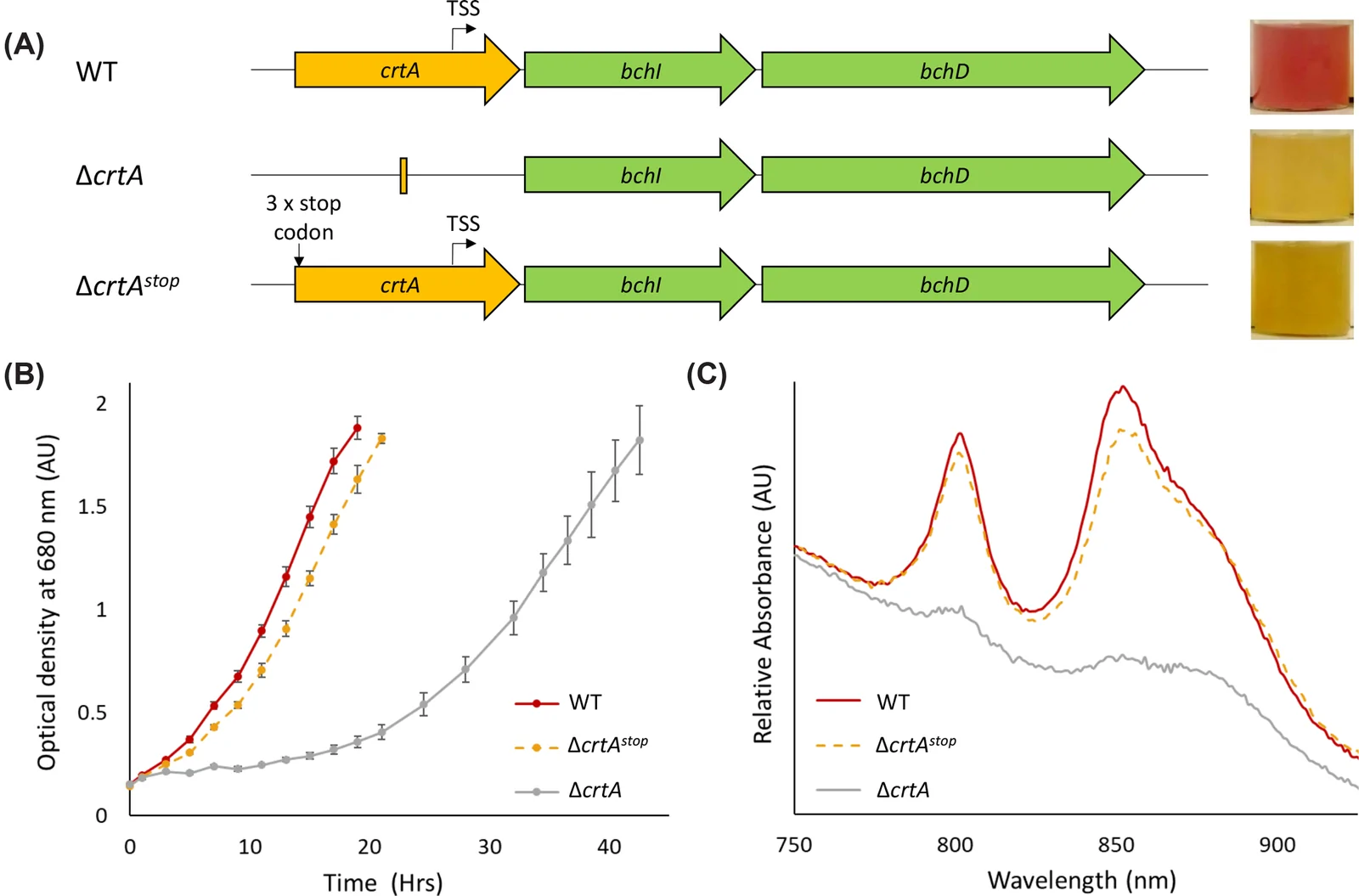
Comparison of ∆*crtA* and Δ*crtA*^stop^ strains (**A**) Schematic representation of the *crtA* locus in WT *C. sphaeroides* and the ∆*crtA* and Δ*crtA*^stop^ mutants and an image of a representative semi-aerobic culture of each strain. The transcription start site (TSS) of the downstream *bchI* gene in *crtA* is indicated; note that this is removed upon deletion of almost the entire *crtA* gene in the original ∆*crtA* strain. (**B**) Photoheterotrophic growth of the WT, ∆*crtA* and Δ*crtA*^stop^ strains. The growth of each strain compared to WT after 19 h was statistically significant for both strains (*P***≤**0.0001). (**C**) Whole cell absorption spectra normalised to cell density (absorbance at 680 nm) of the semi-aerobically grown cultures shown in panel (A) showing the large reduction in photosynthetic complexes in the ∆*crtA* strain.

We previously generated a markerless, in-frame *C. sphaeroides* deletion mutant of *crtA* in which all but the first and last 6 base pairs (bp) of the gene were removed (Figure 2A) [20]. As expected, the resulting Δ*crtA* strain was unable to make spheroidenone, resulting in a yellowish culture as opposed to the deep red colour seen in WT *C. sphaeroides* grown in semi-aerobic conditions (Figure 2A inset panels). This strain displays a marked growth defect under photoheterotrophic conditions (Figure 2B) that can be explained by a significant reduction in the accumulation of photosynthetic complexes in these cells (Figure 2C), although these phenotypes are not obviously a consequence of the altered carotenoid content due to the absence of CrtA.

The *crtA* gene (Rsp_0272) is situated in the photosynthesis gene cluster downstream of *crtI* (encoding phytoene desaturase) and upstream of *bchI* and *bchD* [24,25] (Figure 2A), which encode two subunits of the magnesium chelatase (MgCh) enzyme that catalyses the first dedicated step in BChl biosynthesis [26,27]. The transcription start site (TSS) for *bchID* has been determined to be located 301 bp upstream of the start codon of *bchI*, well within the *crtA* coding sequence [28]. Thus, we hypothesised that the growth phenotype of the Δ*crtA* strain was likely at least in part a consequence of the removal of the TSS resulting in a polar effect of *crtA* deletion on the transcription of the *bchID* genes, affecting BChl biosynthesis and in turn the accumulation of LH2 and RC–LH1 complexes. To test this hypothesis, we determined which phenotypes of the Δ*crtA* mutant could be complemented by the introduction of plasmid-borne copies of *crtA*, *bchID* or all three of these genes. As shown in Supplementary Figure S1, complementing Δ*crtA* with *crtA* restored the ability of the cells to make spheroidenone but did not restore WT levels of the RC–LH1 and LH2 complexes. On the other hand, complementation with *bchID* restored the accumulation of photosynthetic complexes to WT levels. Finally, complementation with all three genes restored both spheroidenone biosynthesis and normal levels of photosynthetic complexes. These results confirmed our hypothesis that decreased amounts of complexes in the Δ*crtA* strain is due to the almost complete removal of the *crtA* gene negatively affecting the expression of *bchID*.

To inactivate *crtA* without affecting expression of the downstream *bchID* genes, we generated a strain with three stop codons inserted immediately after the start codon of the *crtA* gene but leaving the rest of the gene intact in the genome (Figure 2A). Absorption spectra showed that this new Δ*crtA*^stop^ strain accumulates spheroidene under semi-aerobic conditions (Figure 2A inset panels) but has approximately WT levels of RC–LH1 and LH2 complexes (Figure 2C), suggesting *bchID* expression was unaffected. Photoheterotrophic growth of Δ*crtA*^stop^ was reduced only slightly compared to the isogenic WT parent strain (Figure 2B), which will be discussed later.

### Structural modelling and sequential truncation of CrtA reveals that the extended C-terminus is not required for spheroidene monooxygenase activity

**Figure 3:**
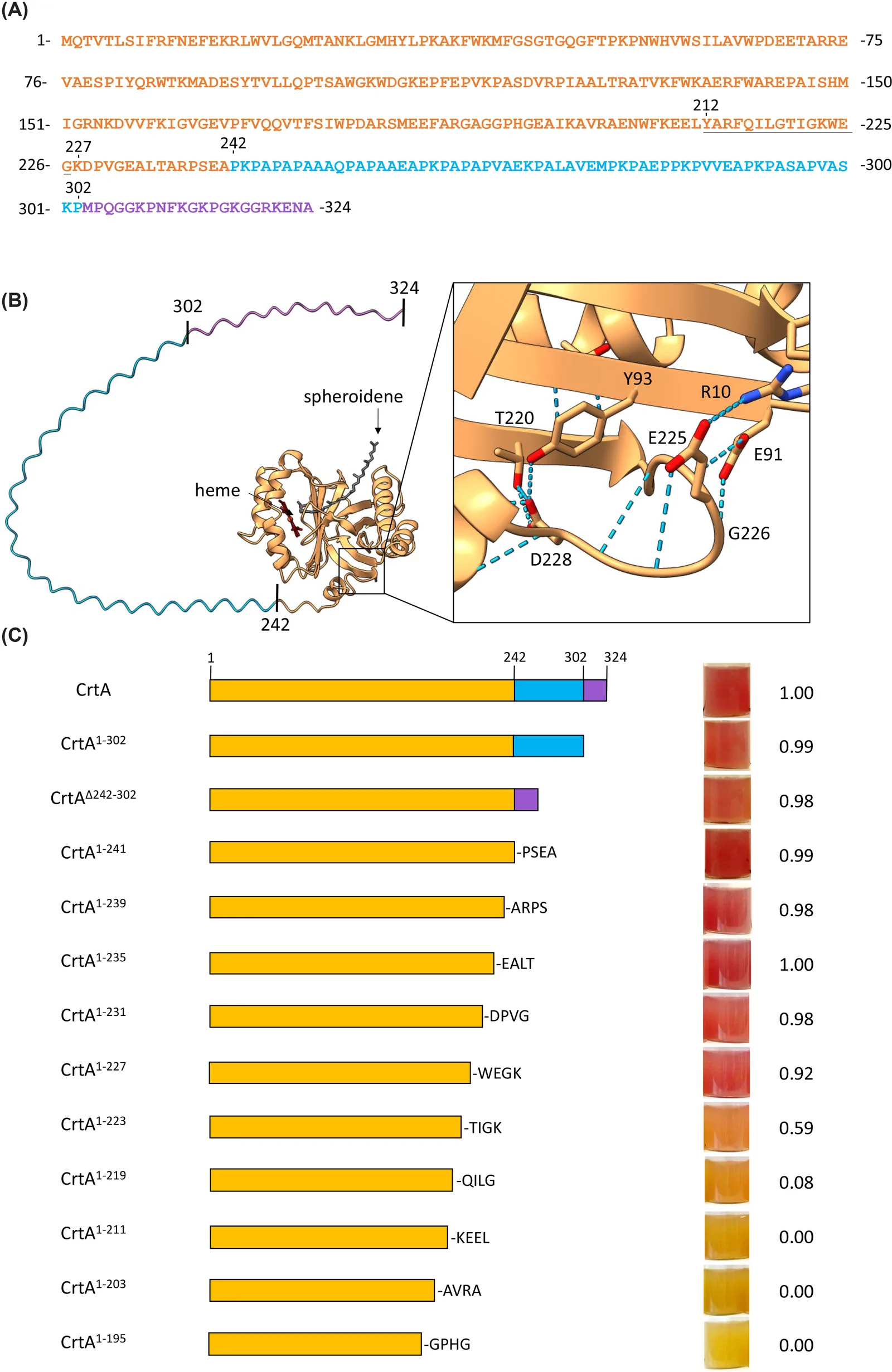
Sequential truncations of the C-terminus of CrtA (**A**) Amino acid sequence of CrtA. The monooxygenase core of the enzyme (residues 1–241) is represented in orange, the proline-rich repeat region (242–302) in blue and the charged C-terminal domain (303–324) in purple. Residues 212–227 are underlined to highlight the region in which truncations begin to lower spheroidenone production. (**B**) AF3 model of *C. sphaeroides* CrtA with heme (maroon) and spheroidene (grey). The structure is shown in the same colour scheme as panel (A). A zoomed-in view of the boxed region with the hydrogen bonds (in blue) formed by residues 220–228 is shown in the inset panel to the right. (**C**) Schematic representation of truncated CrtA enzymes produced in the Δ*crtA*^stop^ mutant background next to an image of a representative semi-aerobic culture of each strain. The sequence is represented in the same colour scheme as panel (A). The number next to the culture indicates the HPLC-determined spheroidenone:spheroidene ratio (see Supplementary Figure S5 for full carotenoid analysis). The last four residues of each sequence are shown at the C-terminus.

An experimentally determined structure of CrtA has yet to be reported and we were unable to crystallise the *C. sphaeroides* enzyme in the present work (see the ‘Discussion’ section for more details), so we generated a structural model of the enzyme using AlphaFold 3 (AF3) [29] in the presence of heme and spheroidene, which is presented in Figure 3. The overall structure is comparable to cofactor-independent antibiotic biosynthesis monooxygenases [30], resembling a C2 pseudo-symmetric alpha-beta barrel, with the alpha helices surrounding the internal beta barrel. Foldseek [31] identified several other proteins with comparable folds to CrtA, many of which are deposited in the Protein Data Bank (PDB; https://www.rcsb.org/) but lack an associated publication (e.g. PDB: 3MCS and 4FVC). In addition to the aforementioned non-heme antibiotic biosynthesis monooxygenase (PDB: 3KG1), Foldseek identified three proteins with similar folds that also bind heme. In just these few examples it is apparent that there is marked variation in activity despite the enzymes adopting comparable folds. Aldoxime dehydratase (PDB: 3A15), which converts aldoximes into the corresponding nitriles with water as the byproduct [32], has a root mean square deviation (RMSD) with the predicted CrtA model of 1.2 Å but very low sequence identity (∼1%). Another enzyme with a comparable fold (RMSD = 1.1 Å) and bound heme is an IsdG-type protein, IsdI (PDB: 3LGM). IsdG and IsdI are non-canonical heme oxygenases that cleave bound heme producing a novel chromophore distinct from biliverdin [33]. The third protein is the oxidative coproheme decarboxylase HemQ (PDB: 5LOQ; RMSD = 1.2 Å); however, as with the IsdG-family proteins, here the substrate acts as the cofactor, generating hydrogen peroxide that carries out the decarboxylation [34].

The 324-residue *C. sphaeroides* CrtA enzyme can be divided into three main regions (Figure 3A,B). Residues 1–241 are predicted to fold into the core of the enzyme, which we predict carries out spheroidene monooxygenase activity (shown in orange in Figure 3A,B). This is followed by a disordered proline-rich sequence containing a high frequency of lysine and glutamate residues interspersed by valine and alanine residues (residues 242–302, shown in blue in Figure 3A,B). Beyond this lies a glycine- and lysine-rich C-terminal region (residues 303–324, shown in purple in Figure 3A,B). An alignment of CrtA sequences from a range of purple bacteria is presented in Supplementary Figure S2. All members of the recently reclassified *Cereibacter* genus contain a similar proline-rich tail at the C-terminus of CrtA. The length of the repeat region varies between 40–93 amino acids, but they share the characteristic repeated proline, lysine and glutamate residues, and contain a number of glycines and lysines within the following C-terminal ∼20 residues.

The AF3 model shows that the interface between the alpha-helical and beta-sheet portion of one pseudo-symmetric unit contains the spheroidene monooxygenase active site (Figure 3B). This contains a heme-binding site formed of a single histidine axial ligand and several hydrophobic residues that form the pocket. Opposing the face coordinated by the axial histidine ligand is a hydrophobic, carotenoid-shaped pore (7-9 Å diameter) that leads out of the protein to the C2 pseudo-symmetric axis. Bordering the pore opening are several hydrophobic residues, which likely form a docking interface with the membrane (discussed later). Rotating the structure 180° from the proposed membrane docking site reveals the location of the large, disordered C-terminal extension. To ascertain the importance of the different regions of CrtA, we generated plasmids encoding a series of increasingly large C-terminal truncations (schematically represented in Figure 3C) and introduced these to the Δ*crtA*^stop^ strain. We measured the carotenoid content of the resulting strains using high-performance liquid chromatography (HPLC) and determined the spheroidenone:spheroidene ratio to see whether the truncated enzymes retained spheroidene monooxygenase activity. We initially introduced plasmids to produce either full-length CrtA, CrtA^1-302^ (truncating the charged C-terminus), CrtA^Δ242-302^ (removing the proline-rich repeat region) or CrtA^1-241^ (truncating both the proline-rich repeat and the charged C-terminus). In all four cases, the enzyme restored the ability of the strains to make spheroidenone, with semi-aerobic cultures having a spheroidenone:spheroidene ratio of between 0.98 and 1.0 (Figure 3C), suggesting that at least the C-terminal 83 residues are not necessary for spheroidene monooxygenase activity.

To ascertain the extent of the C-terminus that could be removed whilst retaining enzymatic activity, we made a series of additional truncations. Deletion of up to 97 C-terminal residues in the strain producing CrtA^1-227^ maintained a spheroidenone:spheroidene ratio of ≥0.92, but removal of only four more amino acids (WEGK) in truncation CrtA^1-223^ lowered this ratio to 0.59. Truncation by another 4 residues (TIGK) to produce CrtA^1-219^ lowered the ratio to just 0.08, with removal of 113 or more residues resulting in a complete absence of spheroidenone in the CrtA^1-211^ strain. The last four residues (224–227) that are required for WT levels of *in vivo* spheroidenone production are found on a loop that leads into the first beta sheet of the inactive half of the pseudo-symmetric protein (Figure 3B inset panel). E225, G226 and D228 are predicted to interact with residues R10, E91 and Y93 of this beta sheet, thus the removal of these residues likely affects the stability of the protein.

Next, plasmids were generated to produce the full-length (CrtA-His) or truncated CrtA^1-241^ (CrtA^1-241^-His) proteins with C-terminal His-tags. The plasmids were introduced to the Δ*crtA*^stop^ background and the His-tagged proteins were isolated on Ni-NTA columns. Complementation with either plasmid returned spheroidenone production to WT levels and western blotting showed similar levels of truncated and full-length CrtA proteins (Supplementary Figure S3). The presence of the His-tag therefore does not affect the enzyme function and the truncation to residue 241 affects neither production nor spheroidene monooxygenase activity. A similar analysis was conducted on His-tagged versions of the truncations where the production of spheroidenone begins to be affected, namely CrtA^1-227^, CrtA^1-223^, CrtA^1-219^, and CrtA^1-211^. Cells containing each of these constructs were normalised to cell density at 680 nm and analysed by SDS–PAGE and immunoblotting. Using CrtA^1-241^-His as a positive control for protein production, we found that all four tested truncations severely lowered CrtA levels, with no detectable signal for CrtA^1-211^ (Supplementary Figure S4). It is possible that interactions further towards the C-terminus are necessary to stabilise the protein, potentially the interaction between D228 and Y93 (Figure 3B inset panel), explaining the reduced protein levels. The complete absence of CrtA^1-211^-His accounts for the abolished production of spheroidenone (Figure 3C); it is interesting to note that the other three truncations, CrtA^1-227^-His, CrtA^1-223^-His and CrtA^1-219^-His, still produce detectable levels of spheroidenone despite the low levels of CrtA.

### Identification of heme and spheroidene interacting residues in CrtA

**Figure 4:**
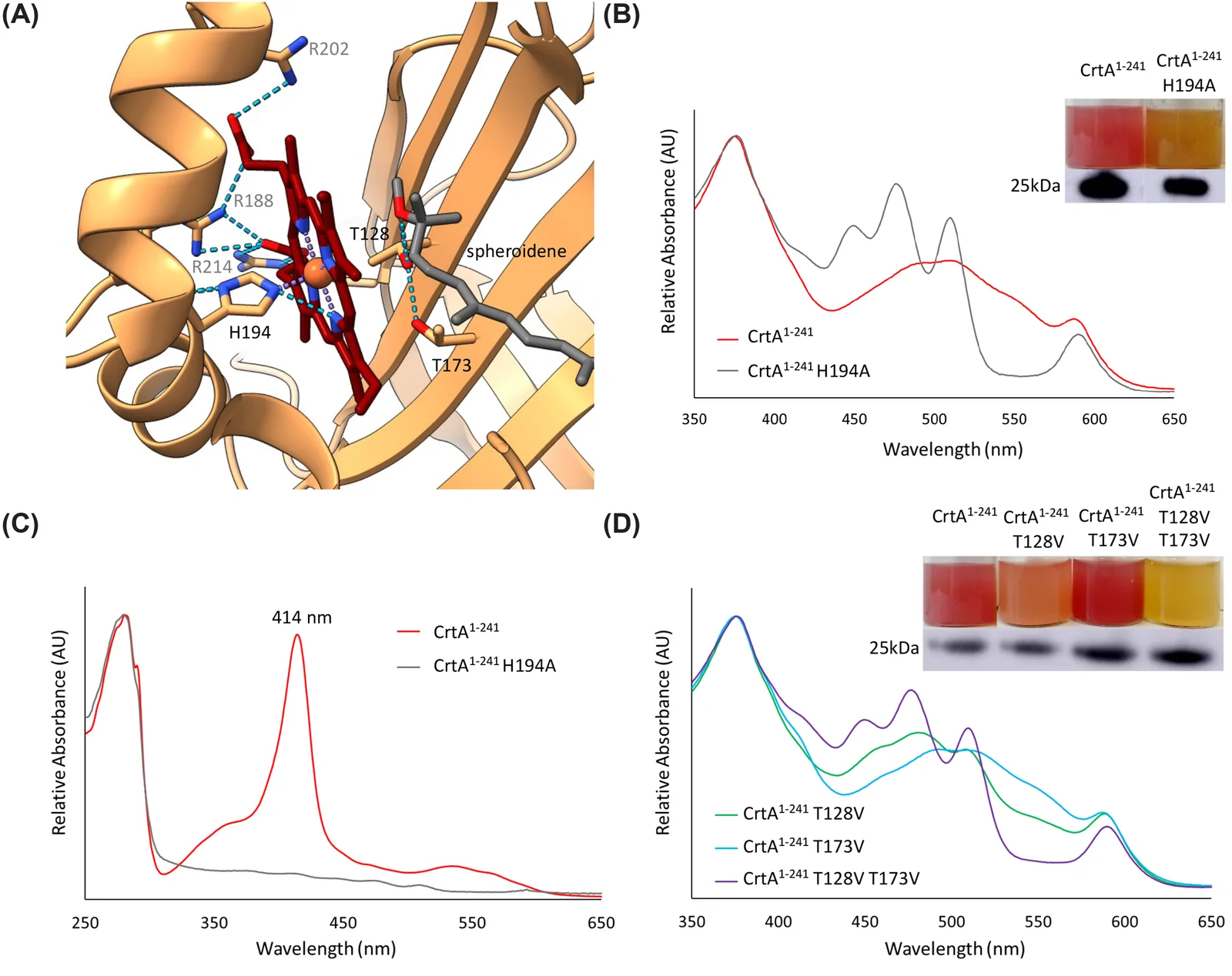
The heme- and substrate-coordinating residues of CrtA (**A**) Visual representation of the AF3 model of the CrtA active site, with H194, T128 and T173 annotated in black, spheroidene shown in grey, and heme in maroon. The positions of the R188, R202 and R214 residues that interact with the propionate side chains of the heme are labelled in light grey. Hydrogen bonds are indicated in blue, and the coordinate bonds of the heme in purple, with the heme iron atom in orange. (**B**) Ultraviolet-visible (UV-Vis) absorbance spectra of ICMs purified from cultures of Δ*crtA*^stop^ complemented with a plasmid containing CrtA^1-241^-His with or without H194 substituted for alanine. The spectra are normalised at 375 nm. The inset shows representative cultures of each strain, with corresponding immunodetection of the His-tag to indicate the levels of CrtA protein. The full blot is shown in Supplementary Figure S6. (**C**) UV-Vis absorbance spectra of CrtA proteins purified from the strains shown in panel (B), normalised at 280 nm. (**D**) UV-Vis absorbance spectra of ICM purified from cultures containing T128V, T173V and T128V T173V variants of CrtA^1-241^-His, normalised at 375 nm. The inset shows representative cultures of each strain, with corresponding immunodetection of the His-tag to indicate the levels of CrtA protein. The full blot is shown in Supplementary Figure S7B.

Previous work with recombinant CrtA has shown it is a heme-containing enzyme [23]. Consistent with this work, our AF3 modelling predicts that H194 acts as the axial ligand to the central Fe atom of a penta-coordinated heme, with the propionate groups hydrogen bonded by three arginine residues, R188, R202 and R214 (Figure 4A). Oxygen is predicted to occupy the opposing coordination site, giving rise to its monooxygenase activity. This histidine was found to be conserved throughout CrtA sequences in closely related species (Supplementary Figure S2). To test the requirement of the heme group for CrtA activity, we introduced the single H194A point mutation into the gene encoding the His-tagged CrtA^1-241^ protein and expressed this gene in the Δ*crtA*^stop^ mutant; unlike the WT enzyme (Figure 4B, red trace and inset panel), the H194A variant did not restore spheroidenone production (Figure 4B, grey trace and inset panel). Whole cells of strains expressing each plasmid were analysed by immunodetection of the His-tag, which showed that similar amounts of WT and H194A CrtA proteins were produced (Figure 4B inset panel and Supplementary Figure S6). Spectra of purified CrtA^1-241^ shows a clear peak corresponding to the heme Soret band at 414 nm with additional smaller peaks at 535 and 565 nm (Figure 4C, red trace), consistent with a previous report of the imidazole-reacted enzyme [23]. These absorption features were absent in the H194A variant of the enzyme, confirming the loss of the heme cofactor when its coordinating histidine residue is changed to alanine (Figure 4C, grey trace) and demonstrating that the heme group is essential for spheroidene monooxygenase activity.

In addition to heme, we also included the spheroidene substrate in our CrtA model. Spheroidene is predicted to enter the putative active site through a channel of hydrophobic residues, with its C2 atom adjacent to the heme group's central iron atom, while the T128 and T173 residues of CrtA interact with the 1-methoxy group and the carotenoid backbone, respectively (Figure 4A). Both these threonine residues are also conserved in the CrtA enzymes of related species (Supplementary Figure S2). To investigate the functions of these residues, we created plasmids to produce T128V and T173V single mutant variants and a T128V T173V double mutant of CrtA^1-241^. The single threonine-substituted enzymes were active, although spheroidenone production was decreased in the CrtA T128V strain, whereas the double variant mainly accumulated spheroidene (Figure 4D). Both the T128V and T128V T173V variants produced small amounts of unidentified carotenoids, highlighted with arrows in Supplementary Figure S7A. Analysis of these strains by western blot revealed similar levels of CrtA proteins, so the effects of the mutations on spheroidenone production can be ascribed to the amino acid substitutions and not decreased levels of the enzymes (Figure 4D inset panel and Supplementary Figure S7B).

### The reduced growth rate of Δ*crtA*^stop^ is due to the lack of the C-terminal tail

**Figure 5:**
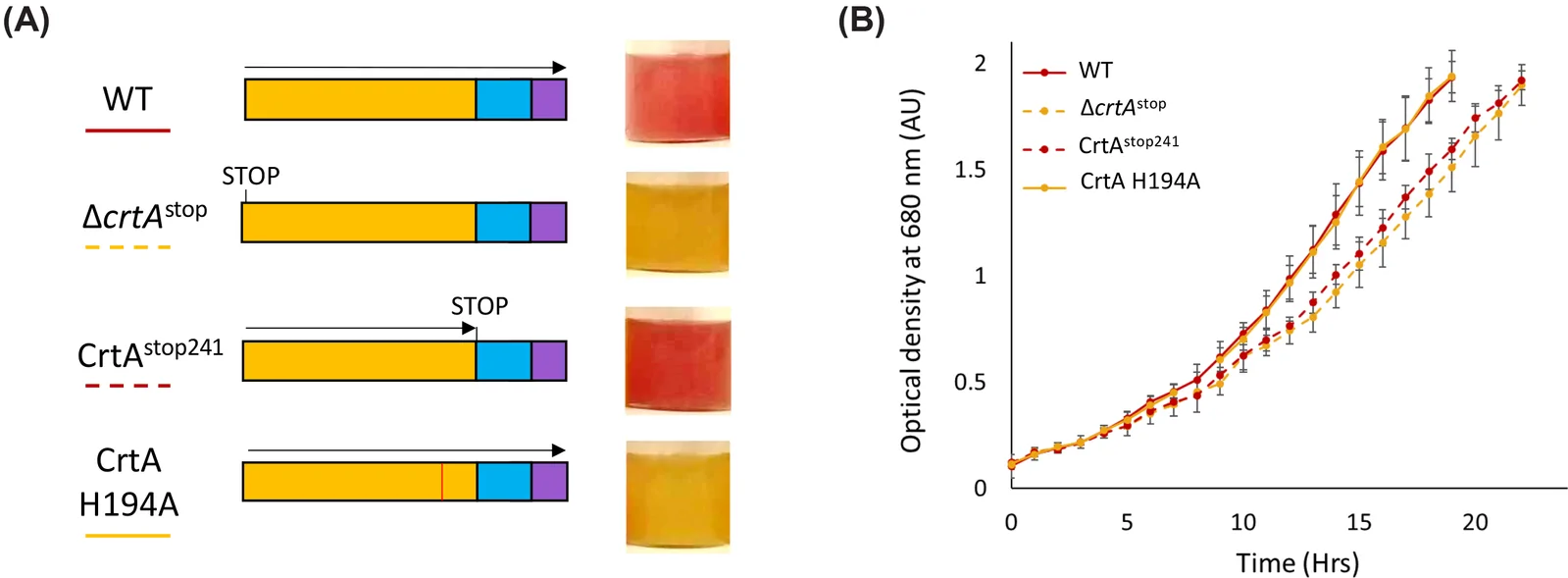
Generation and analysis of strains with genomically modified *crtA* (**A**) Schematic representation of the *crtA* gene in each genomically modified strain, with representative images of cultures of each strain grown in semi-aerobic conditions. The colour scheme corresponds to the encoded regions of the protein, as in Figure 3. The portion of DNA transcribed in each strain is represented with an arrow drawn above, and the position of the H194A mutation is represented with a red line. The line beneath the strain name denotes how it is represented in the growth curve in panel (B). (**B**) Growth curve comparing WT *C. sphaeroides* and the three genomically altered strains described in panel (A) grown with ∼30 μmol photons m^−2^ s^−1^ illumination. Statistical significance of the difference in growth rates was calculated by comparing growth to the WT strain at 18 h. The difference in growth rate relative to the WT was found to be statistically significantly for Δ*crtA*^stop^ (*P*≤0.0002) and CrtA^stop241^ (*P*≤0.0004) but not for CrtA H194A, which almost exactly mimicked WT growth (*P* = 0.503).

To investigate the minor but statistically significant decrease in photoheterotrophic growth of Δ*crtA*^stop^, we generated two further genomically modified strains. In the first of these strains (CrtA^stop241^), three stop codons were inserted into *crtA* after the codon for amino acid 241, such that the encoded C-terminally truncated CrtA^1-241^ enzyme could still produce spheroidenone (Figure 5A) but lacks the tail region. In a second strain (CrtA H194A), the H194A point mutation allowed the production of full-length enzyme lacking spheroidene monooxygenase activity; as expected this strain accumulated spheroidene not spheroidenone (Figure 5A). The Δ*crtA*^stop^ and CrtA^stop241^ strains showed a small but significant decrease in photoheterotrophic growth compared to the WT (*P***≤**0.0002 and *P***≤**0.0004, respectively), whereas growth of CrtA H194A was not significantly different to the WT (*P* = 0.5) (Figure 5B). These results suggest that it is the loss of the C-terminal portion of CrtA that is responsible for the growth defect in Δ*crtA*^stop^ under these conditions rather than the altered carotenoid content. Plasmid-based complementation of Δ*crtA*^stop^ gave the same result (Supplementary Figure S8), with full-length CrtA again restoring WT growth levels, whereas the strain producing the truncated CrtA^1-241^ protein still grew like the Δ*crtA*^stop^ mutant.

### Truncation of CrtA does not prevent its localisation to the ICM

**Figure 6:**
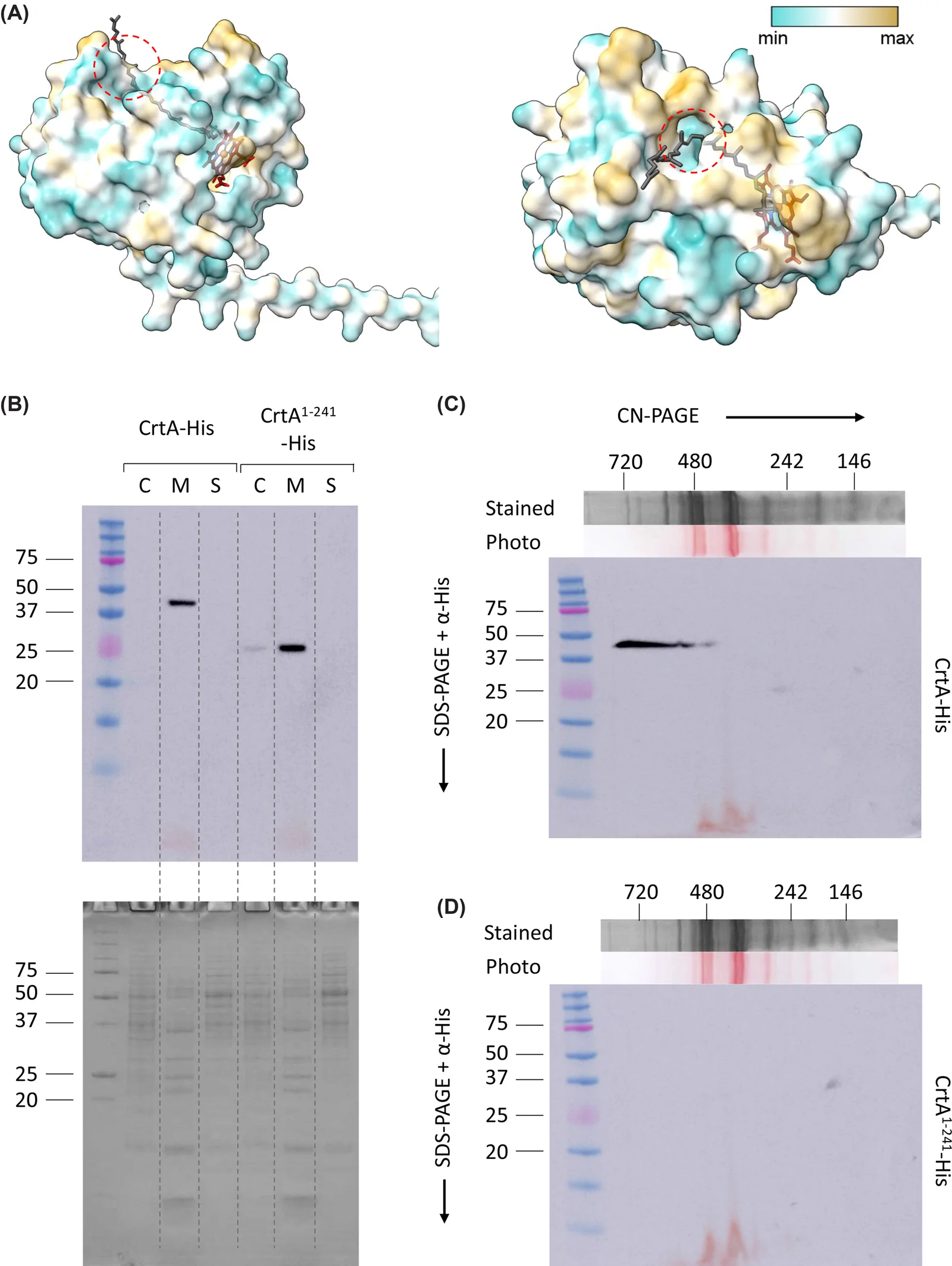
The C-terminal extension mediates CrtA interactions with high-molecular-weight complexes in the membrane (**A**) AF3 model of CrtA with heme (maroon) and spheroidene (grey) with the protein coloured by its surface hydrophobicity (gold, hydrophobic; blue, hydrophilic). A proposed entry point for the substrate flanked by hydrophobic residues creates a face for the enzyme to interact with the surface of the ICM, as shown with the proposed membrane interface pointing uppermost (left) or looking down from the membrane surface (right). The proposed pore is indicated with a dashed red circle. (**B**) Immunodetection of genomically encoded CrtA-His or CrtA^1-241^-His. C denotes cells, M purified membranes, and S soluble fractions. Loading was normalised by protein concentration. Expected molecular weights are 37 kDa for full-length CrtA and 27 kDa for the truncated protein. **(C,D)** 4%-16% Bis-Tris native gel of solubilised ICMs from cells producing full-length CrtA-His (**C**) or truncated CrtA^1-241^-His (**D**). In each case a lane excised from the CN gel was further analysed in the second dimension by SDS–PAGE and the C-terminal His-tag of CrtA was detected by immunoblotting. Images of a replicate CN-PAGE lane before (photo) and after (stained) staining with Coomassie Brilliant Blue are also shown. The numbers to the left of/above the blots/gel in panels (B)–(D) represent the molecular weight in kDa. Stained SDS–PAGE gels corresponding to the blots in panels (C) and (D) are provided in Supplementary Figure S9.

Further analysis of the AF3 model of CrtA reveals a hydrophobic face where the spheroidene substrate protrudes from the enzyme, which could provide an interface with the membrane surface (Figure 6A). We produced genomically modified strains with a His-tag either on the C-terminus of CrtA or inserted before the stop codon in CrtA^stop241^ and confirmed that CrtA was produced at approximately similar levels and localised to the membrane as opposed to the soluble fraction in both cases (Figure 6B). ICMs isolated from both strains grown under semi-aerobic conditions were solubilised in 3% (w/v) n-Dodecyl-β-d-maltoside (β-DDM) and analysed by two-dimensional gel electrophoresis (Figure 6C,D). Clear native (CN)-PAGE was used for separation in the first dimension, with major pigmented bands corresponding to RC-LH1 dimers and monomers and LH2 present at approximately similar levels in both strains. The gel lanes from CN-PAGE were excised and separated in the second dimension by SDS–PAGE, and CrtA was identified by immunodetection of the His-tag. The full-length enzyme formed a high-molecular-weight smear (Figure 6C), suggesting it interacts with a variety of large membrane protein complexes and/or is part of a large complex of proteins and/or self-aggregates. The truncated enzyme did not produce a similar high-molecular-weight smear (Figure 6D), indicating that the C-terminal tail mediates interactions with membrane protein complexes or protein aggregation.

## Discussion

### Structural modelling predicts CrtA is a multidomain protein with an unstructured C-terminus

We have used a combination of mutagenesis, biochemical and modelling approaches to dissect the function of the *C. sphaeroides* spheroidene monooxygenase (CrtA), which catalyses the asymmetric ketolation of the C2 group of spheroidene to generate spheroidenone in the final step of carotenoid biosynthesis in PNSB [16,18]. We were unable to generate crystals of full-length or truncated versions of CrtA using the enzyme purified from *C. sphaeroides* or produced recombinantly in *Escherichia coli*, and with a predicted molecular weight of ∼35.7 kDa the isolated protein is too small for structure determination using cryogenic electron microscopy. Thus, in the absence of an experimentally determined structure, we used AF3 to generate a model of the 324-amino acid enzyme (Figure 3B), which predicted that it adopts a core monooxygenase fold (residues 1–241) followed by a flexible and unstructured 83-residue C-terminal extension consisting of a proline-rich repeat region (242–302) and a charged C-terminal domain (303–324). We showed that this C-terminal extension is not required for spheroidenone production, with a strain producing a truncated enzyme lacking residues 242–324 synthesising WT levels of spheroidene (Figure 3C). CrtA is unique to the PNSB [16] and the C-terminal extension appears to be largely conserved in homologues from the *Cereibacter* genus, into which *C. sphaeroides* was recently re-classified [35], albeit with variation in its length (Supplementary Figure S2). However, this extension is not present in CrtA from *Rba. capsulatus* and is also absent in the enzyme from *Rvi*. *gelatinosus* (Supplementary Figure S2), which produces both spheroidene and spirilloxanthin pathway carotenoids [17,36] and is proposed to have acquired *crtA* by horizontal gene transfer from Bacteroidota [37], where CrtA is a carotenoid 2-hydroxylase rather than a ketolase [38].

### Experimental determination of key residues in the active site of CrtA

Previous *in vitro* characterisation of the *C. sphaeroides* CrtA showed it binds a heme cofactor [23] and modelling the enzyme in the presence of heme and spheroidene allowed us to predict the involvement of H194 in axial heme ligation (Figure 4B) and two threonine residues (T128 and T173) in substrate binding (Figure 4D), which was supported by our experimental evidence presented in this paper. The model further predicted that three arginines (R188, R202 and R214) interact with the heme propionate groups (Figure 4A), although the importance of these residues for activity was not experimentally confirmed here. In addition to molecular oxygen [39], the *Rba. capsulatus* and *Rvi. gelatinosus* CrtA enzymes required an unidentified *E. coli* protein or reduced ferredoxin for *in vitro* monooxygenase activity [15]. We modelled *C. sphaeroides* CrtA with a native *C. sphaeroides* ferredoxin (Rsp_1751), which predicted an interaction such that the [2Fe-2S] cluster of Rsp_1751 is positioned ∼19 Å from the heme in CrtA (Supplementary Figure S10D), but the confidence score for the interaction of these two proteins was low (Supplementary Figure S10E,F). Conversely, NADPH was predicted to occupy a binding pocket in *C. sphaeroides* CrtA on the opposite side of the heme to the carotenoid with higher confidence (Supplementary Figure S10A–C). However, NADPH did not support ketolation by CrtA in previous *in vitro* assays with the *Rba. capsulatus* and *Rvi. gelatinosus* enzymes [15], thus further work is required to clarify the *in vivo* electron donor to *C. sphaeroides* CrtA.

### The C-terminal extension of CrtA is required for optimal photoheterotrophic growth and appears to mediate interactions with complexes in the ICM

We found that our Δ*crtA*^stop^ mutant had a small but reproducible photoheterotrophic growth defect (Figure 2B) but showed that this phenotype was not due to its inability to synthesise spheroidenone (Figure 5B). This is not surprising as CrtA requires oxygen for activity [15] and under anaerobic conditions WT *C. sphaeroides* accumulates mainly spheroidene [40]. Production of spheroidenone is predicted to be a protective measure against phototoxicity caused by the combination of oxic conditions and light [19], thus the spheroidene monooxygenase activity of CrtA is expected to be more important in the presence of oxygen and illumination. We showed that the photoheterotrophic growth phenotype of the Δ*crtA*^stop^ mutant was instead due to the absence of the C-terminal portion of the enzyme. CrtA^stop241^ grows slightly more slowly than the WT, as does Δ*crtA*^stop^, which lacks CrtA altogether (Figure 5B). Since CrtA^stop241^ can make spheroidenone and Δ*crtA*^stop^ cannot, and the difference between CrtA^stop241^ and the WT strain is the absence/presence of the C-terminal extension, this suggests that the tail is responsible for this subtle growth phenotype. CrtA therefore appears to have another function, adding to its photoprotective role of producing carotenoids capable of quenching singlet oxygen [19]. We further showed that the C-terminal tail seems to increase the affinity of CrtA for other proteins present in the ICM, (Figure 6C), which is not the case for the truncated enzyme lacking the tail (Figure 6D).

CrtA is predicted to lack transmembrane helices so it is not expected to be an integral membrane protein, yet we found it localises exclusively with the ICM fraction (Figure 6B). We identified a potential hydrophobic membrane interface in the core monooxygenase region of the enzyme (Figure 6A), which appears to be sufficient for interaction with the ICM, as the truncated version of CrtA lacking the C-terminal extension still localised to the membrane fraction (Figure 6B). The position of this putative membrane interface adjacent to an apparent cavity would appear to allow the highly hydrophobic carotenoid substrate and product access to and from the active site of the enzyme. A membrane location could also promote proximity to other enzymes involved in carotenoid biosynthesis as well as the assembly machinery for the LH2 and RC–LH1 complexes, although a major role in these processes seems unlikely given only the small growth defect in the absence of CrtA.

The way in which the C-terminal tail of CrtA promotes associations with complexes in the ICM, and its functional significance, are not known. AF3 predicts the tail to be disordered (Supplementary Figure S10B), but it may become structured when CrtA interacts with the membrane or proteins. Proteins containing regions of intrinsic disorder have been found to be involved in a variety of signalling, regulation and control pathways [41,42]. For example, RNase E has been found to contain binding sites for three components of the degradosome within its proline-rich C-terminus [43], whilst Khemici et al [44] discovered that a section of this tail is necessary for association of the enzyme with the membrane. The C-terminal tail of *C. sphaeroides* CrtA lacks the characteristic arginine-rich patches of RNaseE that bind to the other proteins in the degradosome and is significantly smaller, so it seems unlikely that it organises the other carotenoid enzymes, but this cannot be ruled out at this stage. We did not identify any specific CrtA binding partners using the His-tagged protein as bait, although CrtA was previously identified in co-immunoprecipitations of the LH1 assembly factor LhaA [45]. Elucidation of CrtA interaction partners should be a target for future studies, although given the length and highly charged nature of the tail it may use weak electrostatic charges to briefly and reversibly bind to a variety of different proteins in a more promiscuous and/or transient fashion. Further work is also needed to clarify the function of CrtA beyond spheroidenone production and to determine how the C-terminal tail mediates interactions with protein complexes in the membrane.

## Materials and methods

### Generation of strains and plasmids

*Cereibacter sphaeroides* strains used in the present study are described in Table 1. Genomic modifications were made using the pK18mob*sacB* plasmid [46]. Briefly, the target genes were deleted by amplifying ∼400 bp regions upstream and downstream of the target sequence and joining them by overlap extension PCR, yielding the desired sequence with adequately sized flanking regions to recombine in the genome [47]. To introduce a premature stop codon to prevent expression of *crtA* (Rsp_0272; Uniprot accession: Q3J189), the sequence TGATAGTGA was inserted directly after the start codon. The resulting PCR fragments were digested with EcoRI and HindIII and ligated into pK18mob*sacB* cut with the same restriction enzymes. Ligation reactions were transformed into *E. coli* JM109 (Promega) and the resulting sequence-verified (Eurofins Genomics or Plasmidsaurus) plasmids were transformed into *E. coli* S17-1 [48] for conjugation into *C. sphaeroides*. Correctly modified strains were isolated following sequential selection with kanamycin (30 μg ml^−1^) and counter-selection with sucrose (10% w/v), and the modified loci were verified by PCR and Sanger sequencing (Eurofins Genomics). Full lists of plasmids and primers used in the present study can be found in Supplementary Tables S1 and S2.

**Table 1:** Strains of *C. sphaeroides* used in the present study

Strain name	Description	Source/reference
WT	Strain 2.4.1	S. Kaplan, The University of Texas Medical School
∆*crtA*	Deletion of *crtA* (rsp_0272) lacking 963 out of 975 bp; carotenoid pathway truncated at spheroidene.	Chi et al. 2015 [20]
Δ*crtA*^stop^	Insertion of TGATAGTGA after the start codon in *crtA* (rsp_0272) in the genome; makes spheroidene.	The present study
CrtA^stop241^	Truncation of *crtA* (rsp_0272) by insertion of TGATAGTGA after codon 241 in the genome; makes spheroidenone.	The present study
CrtA H194A	Site-directed mutant of *crtA* to substitute histidine 194 with alanine; makes spheroidene.	The present study

### Cell growth

Semi-aerobic (microoxic) cultures were grown by inoculating a colony into 10 ml of M22+ media [49] with 1% (w/v) casamino acids (CAA) in a 30 ml universal tube and grown at 30°C with shaking at 180 rpm until visibly pigmented. These starter cultures were used to inoculate 80 ml M22+ with 1% (w/v) CAA in 150 ml conical flasks and subsequently these were used to inoculate 1.6 l of medium in 2.5 l conical flasks. Cells were grown photoheterotrophically in full 1 l Roux bottles containing M22+ medium and 1% (w/v) CAA under ∼30 μmol m^−2^ s^−1^ illumination from 70 W Phillips Halogen Classic bulbs until they reached stationary phase (an optical density at 680 nm (OD_680_) of ∼3). Strains containing pBBRBB plasmids [50,51] were supplemented with 30 μg ml^−1^ kanamycin. Cells were harvested by centrifugation at 4000 RCF for 30 min at 4°C then resuspended in 20 mM Tris pH 8.

For growth curves, strains were grown semi-aerobically to the 80 ml culture stage to an OD_680_ of ∼2. Cells were diluted into full and stoppered 16 ml glass specimen tubes to an OD_680_ of 0.1 and grown under the same illumination conditions as above under 8/16 h day/night cycles, with growth monitored by measuring the OD_680_ using a Biochrom Colourwave CO7000 medical colorimeter.

### Preparation of intracytoplasmic membranes

Following addition of ∼20 mg of DNaseI and lysozyme, cells were broken via two passes through a chilled French press at 18,000 psi followed by removal of unbroken cells and insoluble debris by centrifugation at 25,000 RCF for 30 min at 4°C. The supernatant was layered on top of 15/40% (w/v) discontinuous sucrose gradients and centrifuged at 85,000 RCF in a Beckman Type 45 Ti rotor at 4°C for 10 h. A pigmented band of ICM formed at the 15/40% interface, which was harvested using a serological pipette and stored at −20°C until required.

### Clear native-PAGE analysis of whole complexes

Protein complexes were separated by CN‐PAGE using precast NativePAGE™ Novex^®^ 4%–16% Bis‐Tris gels. The anode buffer was 25 mM imidazole pH 7, and the cathode buffer was 0.03% (w/v) β‐DDM, 0.005% (w/v) deoxycholic acid, 50 mM Tricine, and 7.5 mM imidazole pH 7. Purified membranes with an absorbance at 850 nm of 3 were solubilised in a total volume of 100 μl for 1 h at 4°C in the dark with 3% (w/v) β‐DDM prior to centrifugation at 15,000 rpm for 30 min at room temperature. The insoluble pellet was discarded, and the sample made up to contain 20% (w/v) glycerol with a 50% (w/v) glycerol stock. Twenty-five microlitres of sample was added to each lane and the gel was run at a constant current of 10 mA for 5 h at 4°C. Lanes were excised from native gels with a scalpel and incubated in 1× Laemmli buffer for 10 min. Slices were then transferred into the well of a precast NuPAGE 1× 2D well 12% Bis-Tris gel and run for 60 min at 180 V in NuPAGE™ MES SDS Running Buffer (Invitrogen), before visualisation with Coomassie brilliant blue or transfer for western blot.

### Immunoblotting

Proteins from denaturing gels were transferred to Invitrolon PVDF membranes (Invitrogen) overnight at 4°C at a constant current of 30 mA. After blocking with 5% (w/v) Marvel milk powder for 1 h, blots were incubated with 1:1000 diluted Rabbit anti-6-His Tag primary antibody (Bethyl) for 1 h and washed three times with antibody buffer (0.05% (w/v) TWEEN 20 in 50 mM Tris pH 7.6, 150 mM NaCl). Blots were incubated with 1:10,000 diluted Mouse Anti-Rabbit IgG (γ-chain specific) HRP-conjugated monoclonal secondary antibody (Merck) for 1 h before visualisation with Cyanagen Westar Sun reagent on an Amersham Imager 600.

### Ni-NTA affinity chromatography

Purified membranes with an absorbance of at least 25 at 875 nm were pelleted at 185,000 RCF for 2 h at 4°C and homogenised in 100 μl of 20 mM Tris pH 8. Resuspended membranes with an absorbance of 10 at 875 nm were solubilised in a total volume of 10 ml of 20 mM Tris pH 8, 3% (w/w) β-DDM at 4°C for 1 h. The solubilisation was diluted to 20 ml with loading buffer (20 mM Tris pH 8, 100 mM NaCl, 20 mM imidazole, 0.03% (w/w) β-DDM) and loaded onto a 5 ml chelating Sepharose column (Cytivia) charged with 40 mg ml^−1^ NiSO_4_. The column was washed with 20 ml of loading buffer, then a further 20 ml of wash buffer A (loading buffer but with an increased imidazole concentration of 68 mM) and eluted with 10 ml of elution buffer (loading buffer but with an increased imidazole concentration of 212 mM) before concentrating in a Vivaspin 50 kDa spin concentrator (Sartorius).

### Carotenoid analysis

Samples were prepared from harvested 80 ml semi-aerobic cell pellets resuspended in 1 ml of 20 mM Tris pH 8. Thirty microlitres of resuspended cells were pelleted (15,000 RCF for 5 min at 4°C), the supernatant was completely removed using a pipette, and the pigments were extracted from the pellets by vortexing in 1 ml of methanol in darkness. Following centrifugation (15,000 RCF for 5 min at 4°C), the carotenoids from clarified methanolic pigment extracts were partitioned into 500 μl of hexane. The hexane-pigment layer was carefully isolated, and the solvent was evaporated by centrifuging under vacuum at room temperature using an Eppendorf concentrator. The dried pigments were then resuspended in 300 μl of 90:10 acetonitrile:methanol.

Carotenoids were analysed on an Agilent 1200 high-performance liquid chromatography (HPLC) system, using a Phenomenex C18(2) Luna reverse phase column (5 μm particle size, 100 Å pore size, 250 × 4.6 mm) at a column chamber temperature of 40°C. The column was equilibrated in the mobile phase (90:10 acetonitrile:methanol) prior to sample injection and isocratic separation at a flow rate of 1.2 ml min^−1^ for 50 min. Carotenoid elution was detected using a multichannel diode array detector (Agilent) monitoring absorbance at 475 nm.

### Structural modelling with AlphaFold 3

AF3 was downloaded and installed from the github repository (https://github.com/google-deepmind/alphafold3/blob/main/docs/input.md). All calculations were carried out on Nvidia 80G A100 GPUs. Protein sequences were retrieved from the KEGG database (https://www.kegg.jp/). Ligands were specified using their chemical components dictionary three letter codes as follows: spheroidene (SPO), [2Fe-2S] cluster (FES), heme (HEM), and NADPH (NDP). Multiple sequence alignments were run as per the AF3 data pipeline.

## Supplementary Material

Supplementary Figures S1-S10 and Tables S1-S2

## Data Availability

Any data not provided within the paper as a table, figure, or supplementary file are available from the corresponding authors upon request.

## Open Access

Open access for this article was enabled by the participation of the University of Sheffield in an all-inclusive Read & Publish agreement with Portland Press and the Biochemical Society under an agreement with JISC.

## Abbreviations

β-DDM: N-Dodecyl-β-d-maltoside
AF3: AlphaFold 3
BChl: bacteriochlorophyll
bp: base pair
CAA: casamino acid
Chl: chlorophyll
CN-PAGE: clear native-polyacrylamide gel electrophoresis
HPLC: high-performance liquid chromatography
ICM: intracytoplasmic membrane
LH2: light-harvesting complex 2
PNSB: purple non-sulphur bacteria
*Rba*: *Rhodobacter*
RC-LH1: reaction centre light-harvesting complex 1
*Rvi*: *Rubrivivax*
TSS: transcription start site
UV-Vis: Ultraviolet-visible
WT: wild type

## References

[1] Mirkovic T., Ostroumov E.E., Anna J.M., Van Grondelle R., Govindjee and Scholes G.D. (2017) Light absorption and energy transfer in the antenna complexes of photosynthetic organisms. Chem. Rev. 117, 249–293 10.1021/acs.chemrev.6b00002 27428615

[2] Croce R. and Van Amerongen H. (2014) Natural strategies for photosynthetic light harvesting. Nat. Chem. Biol. 10, 492–501 10.1038/nchembio.1555 24937067

[3] Van Amerongen H. and Croce R. (2025) Nonphotochemical quenching in plants: mechanisms and mysteries. Plant Cell. 37, koaf240 10.1093/plcell/koaf240 41058045 PMC12619064

[4] Lang H.P. and Hunter C.N. (1994) The relationship between carotenoid biosynthesis and the assembly of the light-harvesting LH2 complex in *Rhodobacter sphaeroides*. Biochem. J. 298, 197–205 10.1042/bj2980197 8129720 PMC1138001

[5] Polívka T. and Frank H.A. (2010) Molecular factors controlling photosynthetic light harvesting by carotenoids. Acc. Chem. Res. 43, 1125–1134 10.1021/ar100030m 20446691 PMC2923278

[6] Adams P.G. and Hunter C.N. (2012) Adaptation of intracytoplasmic membranes to altered light intensity in *Rhodobacter sphaeroides*. Biochim. Biophys. Acta 1817, 1616–1627 10.1016/j.bbabio.2012.05.013 22659614

[7] Sener M., Strumpfer J., Singharoy A., Hunter C.N. and Schulten K. (2016) Overall energy conversion efficiency of a photosynthetic vesicle. Elife 5, e09541 10.7554/eLife.09541 27564854 PMC5001839

[8] Qian P., Swainsbury D.J.K., Croll T.I., Castro-Hartmann P., Divitini G., Sader K. et al. (2021) Cryo-EM structure of the *Rhodobacter sphaeroides* light-harvesting 2 complex at 2.1 Å. Biochemistry 60, 3302–3314 10.1021/acs.biochem.1c00576 34699186 PMC8775250

[9] Qian P., Croll T.I., Hitchcock A., Jackson P.J., Salisbury J.H., Castro-Hartmann P. et al. (2021) Cryo-EM structure of the dimeric *Rhodobacter sphaeroides* RC-LH1 core complex at 2.9 Å: the structural basis for dimerisation. Biochem. J. 478, 3923–3937 10.1042/BCJ20210696 34622934 PMC8652583

[10] Qian P., Swainsbury D.J.K., Croll T.I., Salisbury J.H., Martin E.C., Jackson P.J. et al. (2021) Cryo-EM structure of the monomeric *Rhodobacter sphaeroides* RC–LH1 core complex at 2.5 Å. Biochem. J. 478, 3775–3790 10.1042/BCJ20210631 34590677 PMC8589327

[11] Tani K., Kanno R., Kikuchi R., Kawamura S., Nagashima K.V.P., Hall M. et al. (2022) Asymmetric structure of the native *Rhodobacter sphaeroides* dimeric LH1–RC complex. Nat. Commun. 13, 1904 10.1038/s41467-022-29453-8 35393413 PMC8991256

[12] Tani K., Nagashima K.V.P., Kanno R., Kawamura S., Kikuchi R., Hall M. et al. (2021) A previously unrecognized membrane protein in the *Rhodobacter sphaeroides* LH1–RC photocomplex. Nat. Commun. 12, 6300 10.1038/s41467-021-26561-9 34728609 PMC8564508

[13] Cao P., Bracun L., Yamagata A., Christianson B.M., Negami T., Zou B. et al. (2022) Structural basis for the assembly and quinone transport mechanisms of the dimeric photosynthetic RC–LH1 supercomplex. Nat. Commun. 13, 1977 10.1038/s41467-022-29563-3 35418573 PMC9007983

[14] Shneour E.A. (1962) Carotenoid pigment conversion in *Rhodopseudomonas spheroides*. Biochim. Biophys. Acta 62, 534–540 10.1016/0006-3002(62)90235-4 13912201

[15] Gerjets T., Steiger S. and Sandmann G. (2009) Catalytic properties of the expressed acyclic carotenoid 2-ketolases from *Rhodobacter capsulatus* and *Rubrivivax gelatinosus*. Biochim. Biophys. Acta 1791, 125–131 10.1016/j.bbalip.2008.12.006 19136077

[16] Sandmann G. (2023) Genes and pathway reactions related to carotenoid biosynthesis in purple bacteria. Biology 12, 1346 10.3390/biology12101346 37887056 PMC10604819

[17] Takaichi S. and Shimada K. (1999) Pigment composition of two pigment-protein complexes derived from anaerobically and semi-aerobically grown *Rubrivivax gelatinosus*, and identification of a new keto-carotenoid, 2-ketospirilloxanthin. Plant Cell Physiol. 40, 613–617 10.1093/oxfordjournals.pcp.a029584

[18] Takaichi S. (1999) Carotenoids and carotenogenesis in anoxygenic photosynthetic bacteria. The Photochemistry of Carotenoids, pp. 39–69, Springer Netherlands, Dordrecht 10.1007/0-306-48209-6_3

[19] Slouf V., Chabera P., Olsen J.D., Martin E.C., Qian P., Hunter C.N. et al. (2012) Photoprotection in a purple phototrophic bacterium mediated by oxygen-dependent alteration of carotenoid excited-state properties. Proc. Natl. Acad. Sci. U.S.A. 109, 8570–8575 10.1073/pnas.1201413109 22586075 PMC3365192

[20] Chi S.C., Mothersole D.J., Dilbeck P., Niedzwiedzki D.M., Zhang H., Qian P. et al. (2015) Assembly of functional photosystem complexes in *Rhodobacter sphaeroides* incorporating carotenoids from the spirilloxanthin pathway. Biochim. Biophys. Acta 1847, 189–201 10.1016/j.bbabio.2014.10.004 25449968 PMC4331045

[21] Pinta V., Ouchane S., Picaud M., Takaichi S., Astier C. and Reiss-Husson F. (2003) Characterization of unusual hydroxy- and ketocarotenoids in *Rubrivivax gelatinosus:* involvement of enzyme CrtF or CrtA. Arch. Microbiol. 179, 354–362 10.1007/s00203-003-0538-3 12664193

[22] Martin E.C., Bowie A.G., Wellfare Reid T., Hunter C.N., Hitchcock A. and Swainsbury D.J.K. (2024) Sulfoquinovosyl diacylglycerol is required for dimerisation of the *Rhodobacter sphaeroides* reaction centre-light harvesting 1 core complex. Biochem. J. 481, 823–838 10.1042/BCJ20240125 38780411 PMC11346425

[23] Lee P.C., Holtzapple E. and Schmidt-Dannert C. (2010) Novel activity of *Rhodobacter sphaeroides* spheroidene monooxygenase CrtA expressed in *Escherichia coli*. Appl. Environ. Microbiol. 76, 7328–7331 10.1128/AEM.01606-10 20851979 PMC2976243

[24] Lang H.P., Cogdell R.J., Takaichi S. and Hunter C.N. (1995) Complete DNA sequence, specific Tn5 insertion map, and gene assignment of the carotenoid biosynthesis pathway of *Rhodobacter sphaeroides*. J. Bacteriol. 177, 2064–2073 10.1128/jb.177.8.2064-2073.1995 7721699 PMC176850

[25] Naylor G.W., Addlesee H.A., Gibson L.C.D. and Hunter C.N. (1999) The photosynthesis gene cluster of *Rhodobacter sphaeroides*. Photosynth. Res. 62, 121–139 10.1023/A:1006350405674

[26] Gibson L., Willows R.D., Kannangara C.G., von Wettstein D. and Hunter C.N. (1995) Magnesium-protoporphyrin chelatase of *Rhodobacter sphaeroides:* reconstitution of activity by combining the products of the *bchH,-I,* and-*D* genes expressed in *Escherichia coli*. Proc. Natl. Acad. Sci. U.S.A 92, 1941–1944 10.1073/pnas.92.6.1941 7892204 PMC42398

[27] Gibson L.C., Jensen P.E. and Hunter C.N. (1999) Magnesium chelatase from *Rhodobacter sphaeroides:* initial characterization of the enzyme using purified subunits and evidence for a BchI–BchD complex. Biochem. J. 337, 243–251 10.1042/bj3370243 9882621 PMC1219958

[28] Myers K.S., Vera J.M., Lemmer K.C., Linz A.M., Landick R., Noguera D.R. et al. (2020) Genome-wide identification of transcription start sites in two alphaproteobacteria, *Rhodobacter sphaeroides* 2.4.1 and *Novosphingobium aromaticivorans* DSM 12444. Microbial. Res. Announcements 9, e00880–20 10.1128/MRA.00880-20 PMC7471390 32883797

[29] Abramson J., Adler J., Dunger J., Evans R., Green T., Pritzel A. et al. (2024) Accurate structure prediction of biomolecular interactions with AlphaFold 3. Nature 630, 493–500 10.1038/s41586-024-07487-w 38718835 PMC11168924

[30] Grocholski T., Koskiniemi H., Lindqvist Y., Mäntsälä P., Niemi J. and Schneider G. (2010) Crystal structure of the cofactor-independent monooxygenase SnoaB from *Streptomyces nogalater:* implications for the reaction mechanism. Biochemistry 49, 934–944 10.1021/bi901985b 20052967

[31] van Kempen M., Kim S.S., Tumescheit C., Mirdita M., Lee J., Gilchrist C.L.M. et al. (2024) Fast and accurate protein structure search with Foldseek. Nat. Biotechnol. 42, 243–246 10.1038/s41587-023-01773-0 37156916 PMC10869269

[32] Nomura J., Hashimoto H., Ohta T., Hashimoto Y., Wada K., Naruta Y. et al. (2013) Crystal structure of aldoxime dehydratase and its catalytic mechanism involved in carbon-nitrogen triple-bond synthesis. Proc. Natl. Acad. Sci. U.S.A. 110, 2810–2815 10.1073/pnas.1200338110 23382199 PMC3581911

[33] Reniere M.L., Ukpabi G.N., Harry S.R., Stec D.F., Krull R., Wright D.W. et al. (2010) The IsdG-family of haem oxygenases degrades haem to a novel chromophore. Mol. Microbiol. 75, 1529–1538 10.1111/j.1365-2958.2010.07076.x 20180905 PMC3800195

[34] Hofbauer S., Mlynek G., Milazzo L., Pühringer D., Maresch D., Schaffner I. et al. (2016) Hydrogen peroxide-mediated conversion of coproheme to heme *b* by HemQ—lessons from the first crystal structure and kinetic studies. FEBS J. 283, 4386–4401 10.1111/febs.13930 27758026 PMC5157759

[35] Hördt A., López M.G., Meier-Kolthoff J.P., Schleuning M., Weinhold L.M., Tindall B.J. et al. (2020) Analysis of 1,000+ type-strain genomes substantially improves taxonomic classification of Alphaproteobacteria. Front. Microbiol. 11, 468 10.3389/fmicb.2020.00468 32373076 PMC7179689

[36] Harada J., Nagashima K.V.P., Takaichi S., Misawa N., Matsuura K. and Shimada K. (2001) Phytoene desaturase, CrtI, of the purple photosynthetic bacterium, *Rubrivivax gelatinosus*, produces both neurosporene and lycopene. Plant Cell Physiol. 42, 1112–1118 10.1093/pcp/pce140 11673627

[37] Klassen J.L. (2009) Pathway evolution by horizontal transfer and positive selection is accommodated by relaxed negative selection upon upstream pathway genes in purple bacterial carotenoid biosynthesis. J. Bacteriol. 191, 7500–7508 10.1128/JB.01060-09 19820094 PMC2786601

[38] Rählert N., Fraser P.D. and Sandmann G. (2009) A *crtA*-related gene from *Flavobacterium* P99-3 encodes a novel carotenoid 2-hydroxylase involved in myxol biosynthesis. FEBS Lett. 583, 1605–1610 10.1016/j.febslet.2009.04.025 19383499

[39] Shneour E.A. (1962) The source of oxygen in *Rhodopseudomonas spheroides* carotenoid pigment conversion. Biochim. Biophys. Acta 17, 510–511 10.1016/0006-3002(62)90455-9 13988626

[40] Yeliseev A.A., Eraso J.M. and Kaplan S. (1996) Differential carotenoid composition of the B875 and B800-850 photosynthetic antenna complexes in *Rhodobacter sphaeroides* 2.4.1: involvement of spheroidene and spheroidenone in adaptation to changes in light intensity and oxygen availability. J. Bacteriol. 178, 5877–5883 10.1128/jb.178.20.5877-5883.1996 8830681 PMC178441

[41] Cortese M.S., Uversky V.N. and Dunker A.K. (2008) Intrinsic disorder in scaffold proteins: getting more from less. Prog. Biophys. Mol. Biol. 98, 85–106 10.1016/j.pbiomolbio.2008.05.007 18619997 PMC2671330

[42] Williamson M.P. (1994) The structure and function of proline-rich regions in proteins. Biochem. J. 297, 249–260 10.1042/bj2970249 8297327 PMC1137821

[43] Vanzo N.F., Li Y.S., Py B., Blum E., Higgins C.F., Raynal L.C. et al. (1998) Ribonuclease E organizes the protein interactions in the *Escherichia coli* RNA degradosome. Genes Dev. 17, 2770–2781 10.1101/gad.12.17.2770 PMC317140 9732274

[44] Khemici V., Poljak L., Luisi B.F. and Carpousis A.J. (2008) The RNase E of *Escherichia coli* is a membrane‐binding protein. Mol. Microbiol. 70, 799–813 10.1111/j.1365-2958.2008.06454.x 18976283 PMC7610891

[45] Mothersole D.J., Jackson P.J., Vasilev C., Tucker J.D., Brindley A.A., Dickman M.J. et al. (2016) PucC and LhaA direct efficient assembly of the light-harvesting complexes in *Rhodobacter sphaeroides*. Mol. Microbiol. 99, 307–327 10.1111/mmi.13235 26419219 PMC4949548

[46] Schäfer A., Tauch A., Jäger W., Kalinowski J., Thierbach G. and Pühler A. (1994) Small mobilizable multi-purpose cloning vectors derived from the *Escherichia coli* plasmids pK18 and pK19: selection of defined deletions in the chromosome of *Corynebacterium glutamicum*. Gene 145, 69–73 10.1016/0378-1119(94)90324-7 8045426

[47] Sutherland G.A., Qian P., Hunter C.N., Swainsbury D.J.K. and Hitchcock A. (2022) Engineering purple bacterial carotenoid biosynthesis to study the roles of carotenoids in light-harvesting complexes. Methods Enzymol. 674, 137–184 10.1016/bs.mie.2022.04.001 36008006

[48] Simon R., Priefer U. and Pühler A. (1983) A broad host range mobilization system for *in vivo* genetic engineering: transposon mutagenesis in gram negative bacteria. Bio/Technology 1, 784–791 10.1038/nbt1183-784

[49] Hunter C.N. and Turner G. (1988) Transfer of genes coding for apoproteins of reaction centre and light-harvesting LH1 complexes to *Rhodobacter sphaeroides*. J. Gen. Microbiol. 134, 1471–1480 10.1099/00221287-134-6-1471

[50] Tikh I.B., Held M. and Schmidt-Dannert C. (2014) BioBrickTM compatible vector system for protein expression in *Rhodobacter sphaeroides*. Appl. Microbiol. Biotechnol. 98, 3111–3119 10.1007/s00253-014-5527-8 24509770

[51] Bowie A.G., Hitchcock A., Proctor M.S., Martin E.C., Swainsbury D.J.K. and Hunter C.N. (2025) Cyanobacterial redox carriers support photosynthesis in a purple phototrophic bacterium. Biochem. J. 482, 1123–1144 10.1042/BCJ20253114 40663768 PMC12493188

