# Supplementary material for: Molecular dissection of the Cereibacter sphaeroides spheroidene monooxygenase
Source: Biochem J. 2026 Aug 13;483(9):1653–68. doi: 10.1042/BCJ20260276 (PMC13488962; doi:10.1042/BCJ20260276)
Supplement: Supplementary file 1 [file BCJ-2026-0276_supp.pdf]

Supplementary Information for:

Molecular dissection of the *Cereibacter sphaeroides* spheroidene monooxygenase

Elizabeth C. Martin[1], Karolina O. Panas[1], Felix S. Morey-Burrows[1], Jack. H Salisbury[1,3], Joshua R. Barrett[1], George A. Sutherland[1,4], C. Neil Hunter[1]* and Andrew Hitchcock[1,2]*

[1] Plants, Photosynthesis and Soil, School of Bioscience, University of Sheffield, Sheffield, UK.

[2] Molecular Microbiology: Biochemistry to Disease, School of Bioscience, University of Sheffield, Sheffield, UK.

[3] Present affiliation: Medical Research Council Mitochondrial Biology Unit, University of Cambridge, Cambridge, UK.

[4] Present affiliation: Baseimmune Ltd, Apex, 1 Tribeca Walk, London, UK.

*Co-corresponding authors: C. Neil Hunter (c.n.hunter@sheffield.ac.uk) and Andrew Hitchcock (a.hitchcock@sheffield.ac.uk)

Contents

Pages 2-4 – **Table S1. Plasmids used in this study.**

Pages 5-6 – **Table S2. Primers used in this study.**

Page 7 – **Figure S1. Complementation of Δ*crtA* with *crtA* and/or *bchID*.**

Page 8 – **Figure S2. Sequence alignment of CrtA enzymes from representative PPB.**

Page 9 – **Figure S3. Comparison of the purification of full-length and truncated His-tagged CrtA by Ni-NTA affinity chromatography.**

Page 10 – **Figure S4. Western Blot analysis of strains producing truncations of CrtA-His in the Δ*crtA*^{stop} background.**

Page 11 – **Figure S5. HPLC analysis of the strains presented in Figure 3.**

Page 11 – **Figure S6. Western Blot analysis of strains producing wild-type (WT) and H194A variants of CrtA$^{1-241}$-His in Δ*crtA*^{stop} background.**

Page 12 – **Figure S7. Western Blot analysis of point mutations of CrtA$^{1-241}$-His pBBRBB in Δ*crtA*^{stop}.**

Page 13 – **Figure S8. Growth curve comparing Δ*crtA*^{stop} complemented with pBBRBB-pPuc containing full length CrtA or CrtA$^{1-241}$ in ~30 μmol photons m^{-2} s^{-1} light conditions.**

Page 13 – **Figure S9. Coomassie stained blots of identical gels to blots shown in Figure 6C and D.**

Pages 14-15 – **Figure S10. AlphaFold3 modelling of potential reducing agents for monooxygenase activity.**

Table S1. Plasmids used in this study.

Name	Details	Source/reference
pk18mob*sacB*	Allelic exchange vector, kanamycin resistant (Km^R)	Professor Judith Armitage, University of Oxford
pk18mob*sacB*::CrtA STOP	Construct for the prevention of translation of CrtA by insertion of TGATAGTGA after the start codon in *crtA*	This study
pk18mob*sacB*::CrtA T STOP	Construct for production of a truncated from of CrtA by insertion of TGATAGTGA after the codon for residue 241 in *crtA*	This study
pk18mob*sacB*::CrtA H194A	Construct for genomic insertion of H194A into CrtA	This study
pk18mob*sacB*::CrtA T His-STOP	Construct for production of a His-tagged truncated form of CrtA by insertion of CATCACCATCACCATCACTGATAGTGA after the codon for residue 241 in *crtA*	This study
pBBRBB-Ppuf$_{843\text{-}1200}$-*DsRed*	Replicative expression plasmid driving expression of insured genes from a truncated version of the *puf* promoter, Km^R	Addgene.org; [50]
empty pBBRBB-Ppuf$_{843\text{-}1200}$	pBBRBB-Ppuf$_{843\text{-}1200}$ vector with no insert	[51]
pBBRBB-Ppuc-*pucBAC*	Version of pBBRBB-Ppuf$_{843\text{-}1200}$-*DsRed* with *puf* promoter and dsRED replaced with *puc* promoter and *pucBAC*	[22]
empty pBBRBB-Ppuc	pBBRBB-Ppuc vector with no insert	This study
pBBRBB-Ppuf$_{843\text{-}1200}$-*crtA*	Plasmid for expression of *crtA*	This study
pBBRBB-Ppuf$_{843\text{-}1200}$-*bchID*	Plasmid for expression of *bchID*	This study
pBBRBB-Ppuf$_{843\text{-}1200}$-*crtA bchID*	Plasmid for expression of *crtA* and *bchID*	This study

pBBRBB-Ppuf$_{843\text{-}120}$-CrtA$^{1\text{-}241}$	Plasmid for to produce CrtA residues 1-241	This study
pBBRBB-Ppuf$_{843\text{-}1200}$-CrtA$^{\Delta 242\text{-}302}$	Plasmid to produce CrtA residues 1-241 + 302-324	This study
pBBRBB-Ppuf$_{843\text{-}1200}$-CrtA$^{1\text{-}302}$	Plasmid to produce CrtA residues 1-302	This study
pBBRBB-Ppuc-CrtA$^{1\text{-}239}$	Plasmid to produce CrtA residues 1-239	This study
pBBRBB-Ppuc-CrtA$^{1\text{-}235}$	Plasmid to produce CrtA residues 1-235	This study
pBBRBB-Ppuc-CrtA$^{1\text{-}231}$	Plasmid to produce CrtA residues 1-231	This study
pBBRBB-Ppuc-CrtA$^{1\text{-}227}$	Plasmid to produce CrtA residues 1-227	This study
pBBRBB-Ppuc- CrtA$^{1\text{-}223}$	Plasmid to produce CrtA residues 1-223	This study
pBBRBB-Ppuc- CrtA$^{1\text{-}219}$	Plasmid to produce CrtA residues 1-219	This study
pBBRBB-Ppuc- CrtA$^{1\text{-}211}$	Plasmid to produce CrtA residues 1-211	This study
pBBRBB-Ppuc-CrtA$^{1\text{-}203}$	Plasmid to produce CrtA residues 1-203	This study
pBBRBB-Ppuc-CrtA$^{1\text{-}195}$	Plasmid to produce CrtA residues 1-195	This study
pBBRBB-Ppuc-CrtA$^{1\text{-}324}$-His	Plasmid to produce CrtA with C-terminal His-tag	This study
pBBRBB-Ppuc-CrtA$^{1\text{-}214}$-His	Plasmid to produce CrtA residues 1-241 with C-terminal His-tag	This study
pBBRBB-Ppuc-CrtA$^{1\text{-}214}$H194A-His	Plasmid to produce CrtA H194A residues 1-241 with C-terminal His-tag	This study
pBBRBB-Ppuc-CrtA$^{1\text{-}214}$T128V-His	Plasmid to produce CrtA T128V residues 1-241 with C-terminal His-tag	This study
pBBRBB-Ppuc-CrtA$^{1\text{-}214}$T173V-His	Plasmid to produce CrtA T173V residues 1-241 with C-terminal His-tag	This study
pBBRBB-Ppuc-CrtA$^{1\text{-}214}$T128V T183V-His	Plasmid to produce CrtA T173V T128V residues 1-241 with C-terminal His-tag	This study

pBBRBB-Ppuc-CrtA$^{1-227}$-His	Plasmid to produce CrtA residues 1-227 with C-terminal His-tag	This study
pBBRBB-Ppuc- CrtA$^{1-223}$-His	Plasmid to produce CrtA residues 1-223 with C-terminal His-tag	This study
pBBRBB-Ppuc- CrtA$^{1-219}$-His	Plasmid to produce CrtA residues 1-219 with C-terminal His-tag	This study
pBBRBB-Ppuc- CrtA$^{1-211}$-His	Plasmid to produce CrtA residues 1-211 with C-terminal His-tag	This study

Table S2. Primers used in this study. Restriction enzyme recognition sites and sequences encoding His-tags are underlined. Introduced stop codons are shown in bold.

Name	Sequence (5′ → 3′)
CrtA Screen F	CAGGAACTTCTCGTATCCCGCC
CrtA KO UF EcoRI	CCGGAATTCACCTCGTAGAACGGATCGATCG
CrtA KO DR HindIII	CCCCAAGCTTGGTTGACCTCGTCGATATAGAG
CrtA Screen R	CGAGGAGCAGGTCCACGATGTG
CrtA T UF EcoRI	GACTGAATTCCAAATGGGACGGCAAGGAGC
CrtA T DR HindIII	GATCAAGCTTCGTTCGATCATCGTGGTGGAGG
CrtA T Scr F	GCCCCATACAATAGACTGTCTAGTCAGG
CrtA STOP F	GGAGGAATGACATGTGATAGTGACAGACTGTCAC
CrtA STOP R	GTGACAGTCTGTCACTATCACATGTCATTCCTCC
CrtA STOP DR HindIII	GATCAAGCTTCGATCATGTGCGAGATGGCAGG
CrtA STOP Scr R	CAGATCGAGAAGGTAACCTGCTGG
CrtA Rep STOP F	CCTCCGAGGCTTGATAGTGACCGAAACCC
CrtA Rep STOP R	GGGTTTCGGTCACTATCAAGCCTCGGAGG
CrtATHisSTOP F	CATCACCATCACCATCACTGATAGTGACCGAAACCCGCTC
CrtATHisSTOP R	TCACTATCAGTGATGGTGATGGTGATGAGCCTCGGAGGGGCGTG
CrtA F HindIII	GATCAAGCTTATGCAGACTGTCACGCTCAGTATC
CrtA F BglII	GATCAGATCTATGCAGACTGTCACGCTCAGTATC
CrtA R BcuI	GATCACTAGTTCAGGCGTTCTCTTTGCGGC
$CrtA^{1-302}$ R BcuI	GATCACTAGTTCACGGCTTCGAGGCCACGGG
$CrtA^{\Delta 242-302}$ UR	CTTCCCGCCCTGAGCCTCGGAGGGGCGTG
$CrtA^{\Delta 242-302}$ DF	GAGGCTCAGGGCGGGAAGCCGAACTTC
$CrtA^{1-241}$ R BcuI	GATCACTAGTTCAAGCCTCGGAGGGGCGTGCGGTCA
$CrtA^{1-239}$ R BcuI	GTTCACTAGTTCAGGAGGGGCGTGCGGTCAG
$CrtA^{1-235}$ R BcuI	GTTCACTAGTTCAGGTCAGCGCCTCGCCC
$CrtA^{1-231}$ R BcuI	GTTCACTAGTTCAGCCCACCGGGTCCTTGC

CrtA$^{1-227}$ R BcuI	GTTCACTAGTTCACTTGCCCTCCCACTTACCAATCG
CrtA$^{1-223}$ R BcuI	GTTCACTAGTTCACTTACCAATCGTTCCGAGAATCTGGAAG
CrtA$^{1-219}$ R BcuI	GTTCACTAGTTCATCCGAGAATCTGGAAGCGGG
CrtA$^{1-211}$ R BcuI	GTTCACTAGTTCAAAGCTCCTCCTTGAACCAGTTCTC
CrtA$^{1-203}$ R BcuI	GTTCACTAGTTCAGGCGCGCACGGCCTTG
CrtA$^{1-195}$ R BcuI	GTTCACTAGTTCAGCCATGCGGGCCGC
CrtA H194A F	CCCGGCTGGCGAGGCGATCAAG
CrtA H194A R	CTTGATCGCCTCGCCAGCCGGG
CrtA T128V F	GATCGCCGCCCTCGTCCGGGCCAC
CrtA T128V R	GTGGCCCGGACGAGGGCGGCGATC
CrtA T173V F	CCAGCAGGTTGTCTTCTCGATCTG
CrtA T173V R	CAGATCGAGAAGACAACCTGCTGG
CrtA His R XhoI	GATCCTCGAGGGCGTTCTCTTTGCGGC
CrtA T His R XhoI	GATCCTCGAGAGCCTCGGAGGGGCGTG
CrtA$^{1-227}$ His R XhoI	GTTCCTCGAGCTTGCCCTCCCACTTACCAATCG
CrtA$^{1-223}$ His R XhoI	GTTCCTCGAGCTTACCAATCGTTCCGAGAATCTGGAAG
CrtA$^{1-219}$ His R XhoI	GTTCCTCGAGTCCGAGAATCTGGAAGCGGG
CrtA$^{1-211}$ His R XhoI	GTTCCTCGAGAAGCTCCTCCTTGAACCAGTTCTC

A

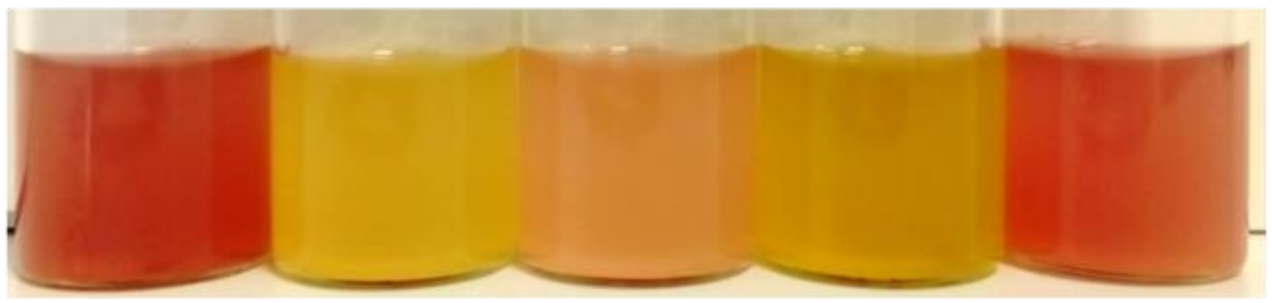

	Empty pBBRBB in WT	Empty pBBRBB in Δ*crtA*	*crtA* pBBRBB in Δ*crtA*	*bchID* pBBRBB in Δ*crtA*	*crtA-bchID* pBBRBB in Δ*crtA*
spheroidenone	95.7	0	100	0	93.9
spheroidene	0	100	0	100	0
neurosporene	4.2	0	0	0	6.1

B

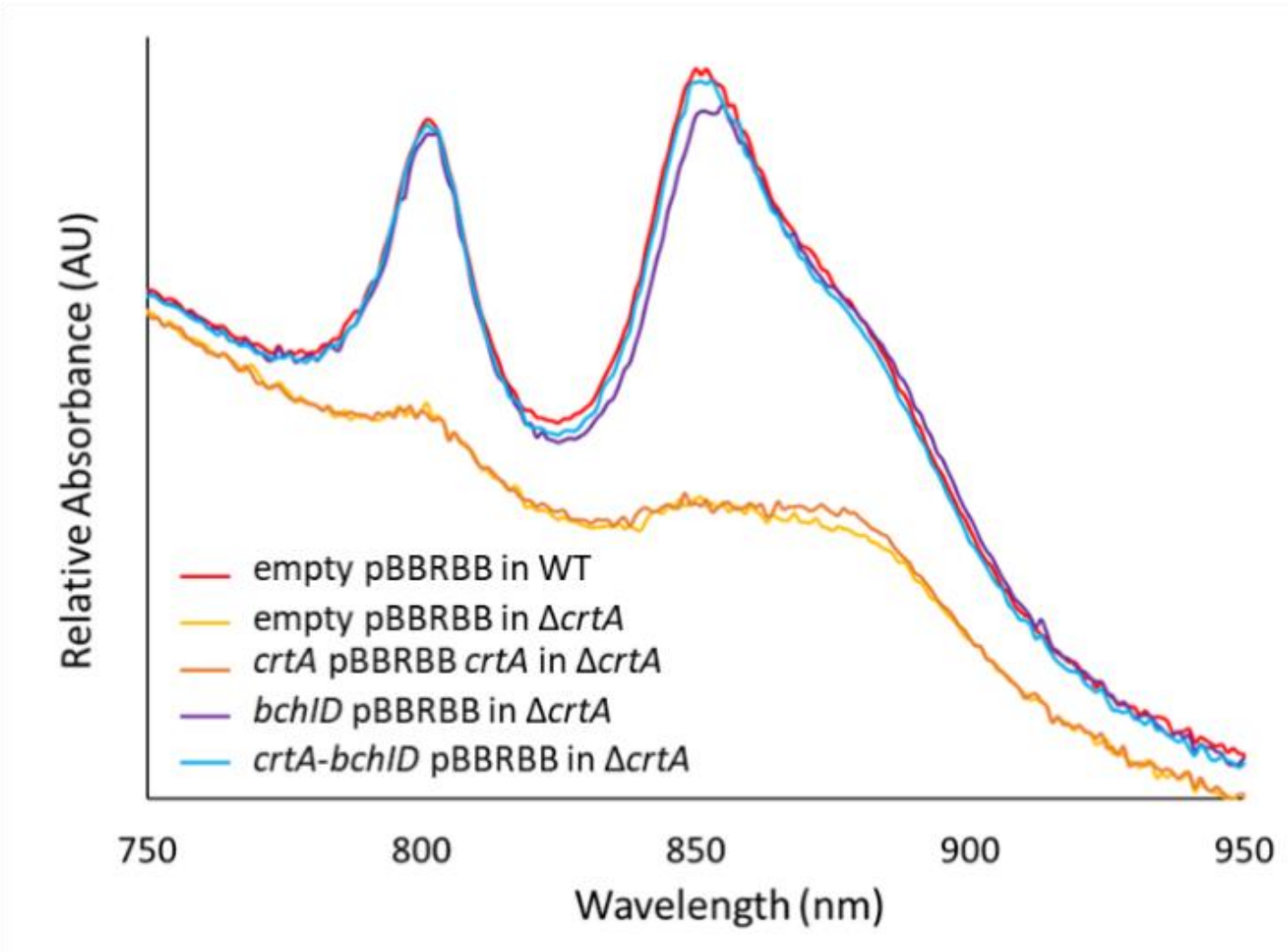

Figure S1. Complementation of Δ*crtA* with *crtA* and/or *bchID*. (A) Photo of 10 mL semi-aerobically grown cells of each control and complemented strain. Plasmids are based on pBBRBB-Ppuf$_{843\text{-}1200}$. HPLC analysis of each strain is shown below as a percentage of total carotenoid content, which was estimated as the area under the peak for each major carotenoid species (also neurosporene) when monitoring absorbance at 475 nm. (B) Absorbance spectra of whole cells from strains shown in (A) normalised to cell density at 680 nm.

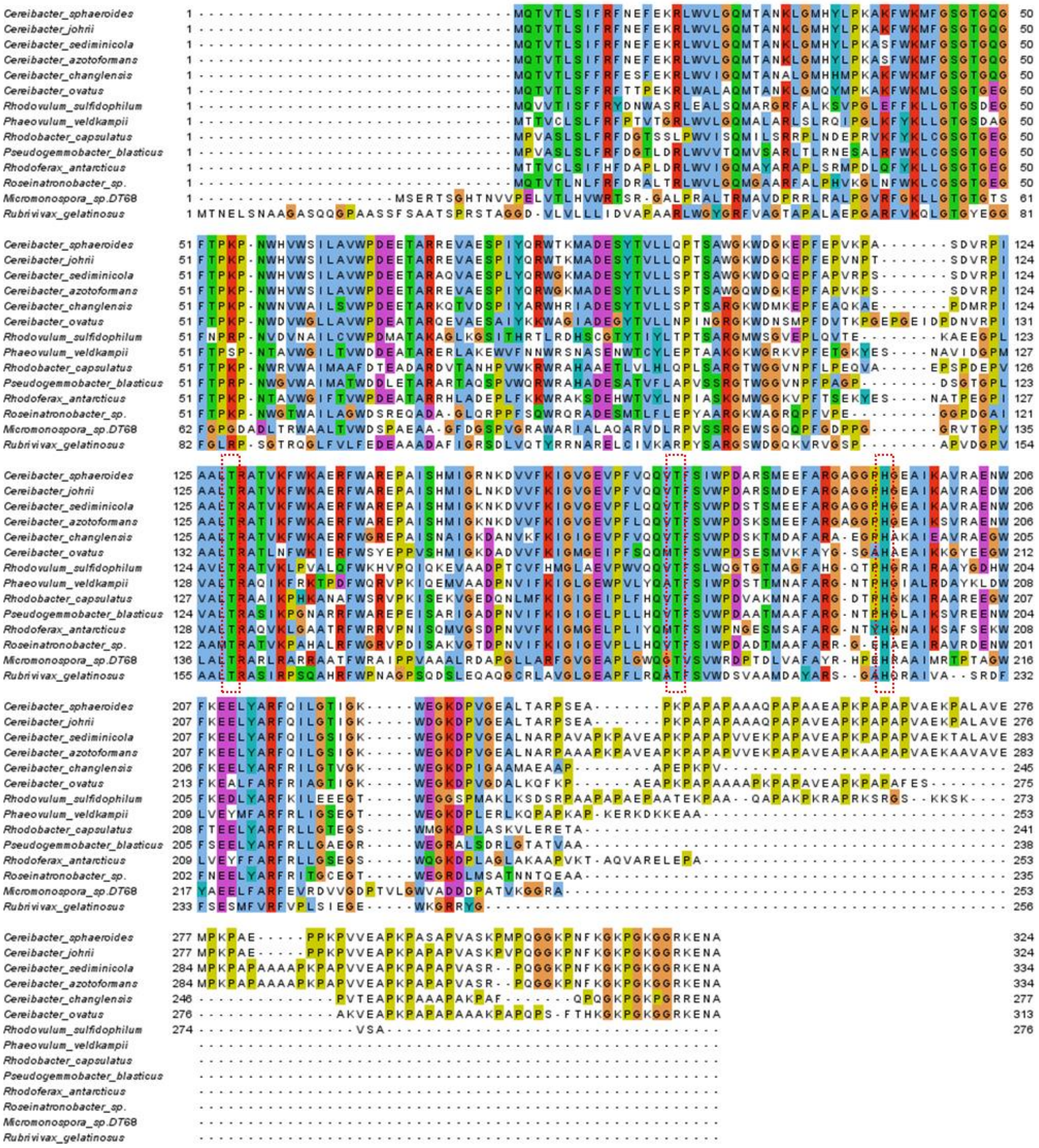

Figure S2. Sequence alignment of CrtA enzymes from representative PPB. Alignment of CrtA homologues identified by a BLAST search (https://blast.ncbi.nlm.nih.gov/Blast.cgi) using the *R. sphaeroides* protein as the query. Conservation of residues shown to be involved in heme or substrate binding in the present study are highlighted with red boxes. NCBI accession codes: *Cereibacter sphaeroides*: WP_002720436, *Cereibacter johrii*: WP_069333038, *Cereibacter sediminicola*: WP_145103434, *Cereibacter azotoformans*: WP_011908062, *Cereibacter changlensis*: WP_107664769, *Cereibacter ovatus*: WP_097030939, *Rhodovulum sulfidophilum*: WP_202259345, *Phaeovulum veldkampii*: WP_235820116, *Rhodobacter capsulatus*: WP_055209574, *Pseudogemmobacter blasticus*: WP_108642787, *Rhodoferax antarcticus*: WP_075586590, *Roseinatronobacter sp.*: WP_296474761, *Micromonospora sp. DT68*: WP_425735504, *Rubrivivax gelatinosus*: WP_014429560.

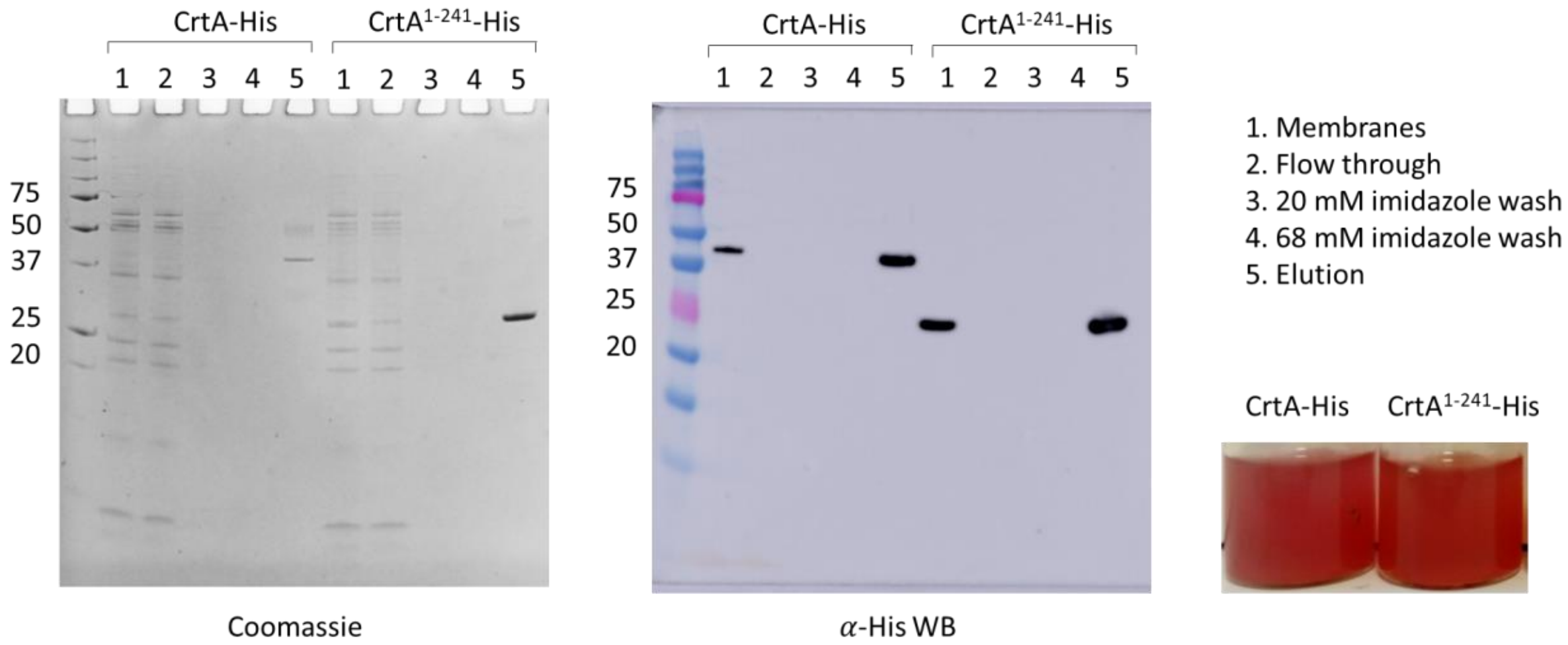

Figure S3. Comparison of the purification of full-length and truncated His-tagged CrtA by Ni-NTA affinity chromatography. Membranes of strains carrying pBBRBB-Ppuc-*crtA*-His or pBBRBB-Ppuc-CrtA$^{1-241}$-His and the subsequent fractions from Ni-NTA affinity chromatography analysed by SDS-PAGE and western blotting. Replica gels were either stained with Coomassie (left) or transferred to membranes for immunodetection of the His-tag (right). Expected molecular weight for full length CrtA is 37 kDa and 27 kDa for the truncated protein. Numbers to the left of the gel/blot indicate the molecular weight (in kDa) of the marker bands in the left-most lane. The inset panel shows representative 10 mL cultures of each strain, showing each makes spheroidenone.

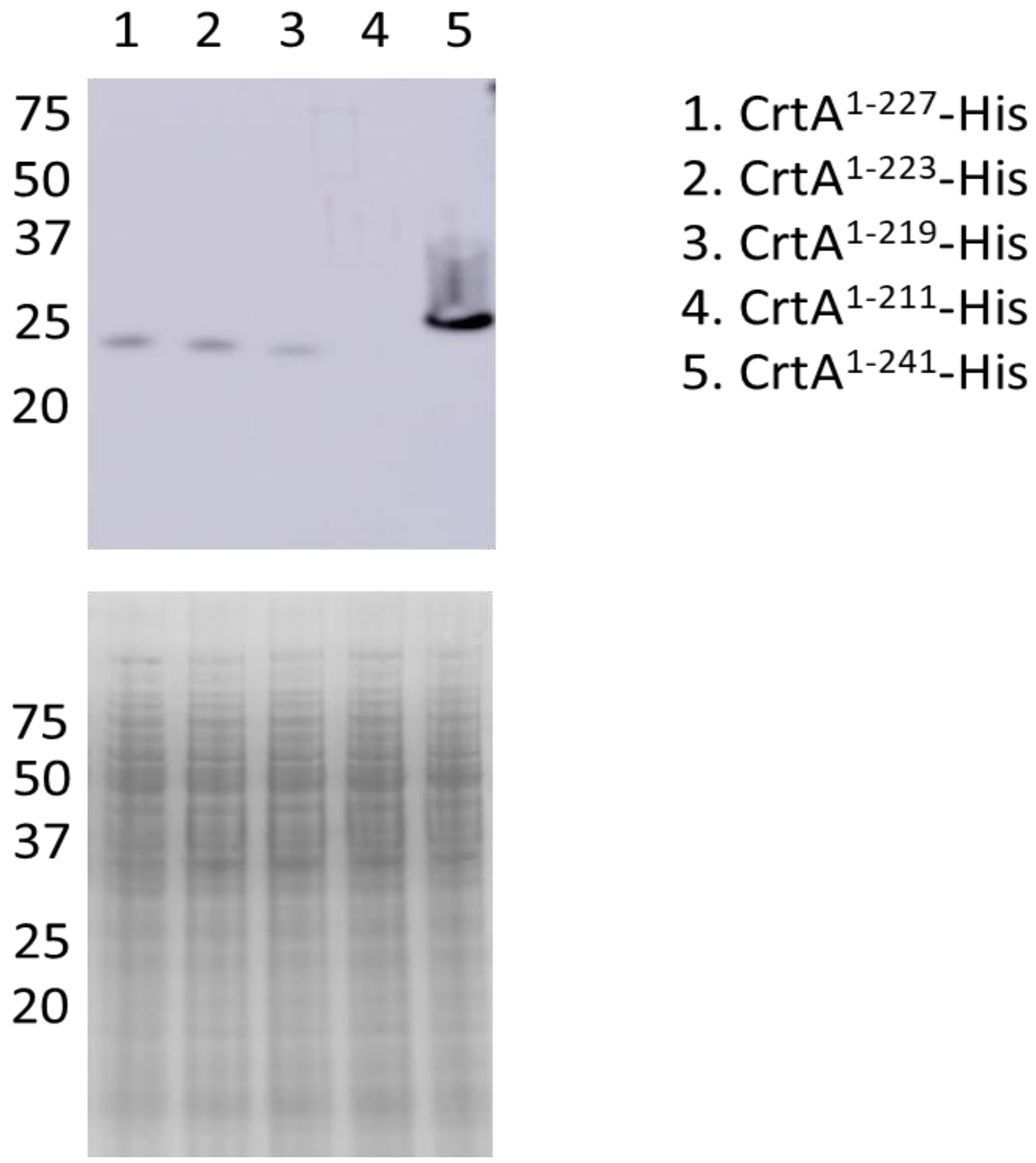

Figure S4. Western Blot analysis of strains producing truncations of CrtA-His in the Δ*crtA*^{stop} background. Whole cell extracts of Δ*crtA*^{stop} containing pBBRBB-Ppuc-CrtA-His to produce His-tagged CrtA truncated to varying degrees (as indicated) analysed by SDS-PAGE and immunoblotting. The gel was stained with Coomassie brilliant blue (below) and an identical gel was transferred to a membrane for immunodetection of the His-tag (above). Loading was normalised to cell density at 680 nm. Numbers to the left of the gel/blot indicate molecular weight in kDa and the expected molecular weight for CrtA$^{1\text{-}241}$-His is 27 kDa.

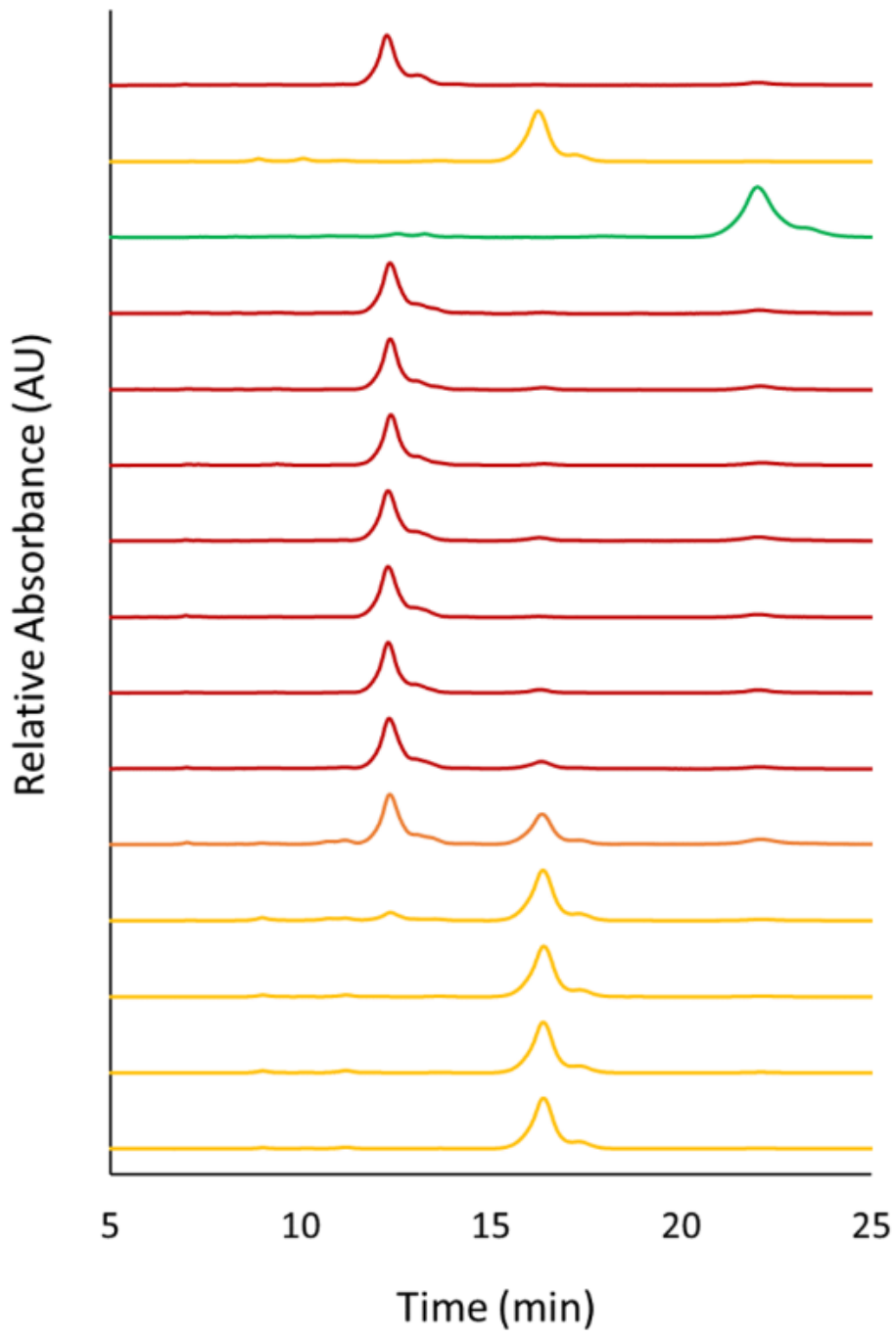

	spheroidenone (%)	spheroidene (%)	neurosporene (%)
WT	98.3	0	1.7
Δ*crtA*^{STOP}	0	100	0
Δ*crtC*	0	0	100
CrtA$^{1-302}$	94.8	0.8	4.4
CrtA$^{\Delta 242-302}$	93.6	1.7	4.7
CrtA$^{1-241}$	96.7	0.9	2.4
CrtA$^{1-239}$	94.9	2.2	2.9
CrtA$^{1-235}$	97.2	0	2.8
CrtA$^{1-231}$	95.6	2.2	2.2
CrtA$^{1-227}$	91.0	8.0	1.0
CrtA$^{1-223}$	57.0	39.0	4.0
CrtA$^{1-219}$	7.9	91.7	0.4
CrtA$^{1-211}$	0	100	0
CrtA$^{1-203}$	0	100	0
CrtA$^{1-195}$	0	100	0

Figure S5. HPLC analysis of the strains presented in Figure 3. As a measure of CrtA activity, the ratio of spheroidene and spheroidenone was determined by calculating their percentage of the total carotenoid content of each strain, which was calculated as described in the legend of Figure S1.

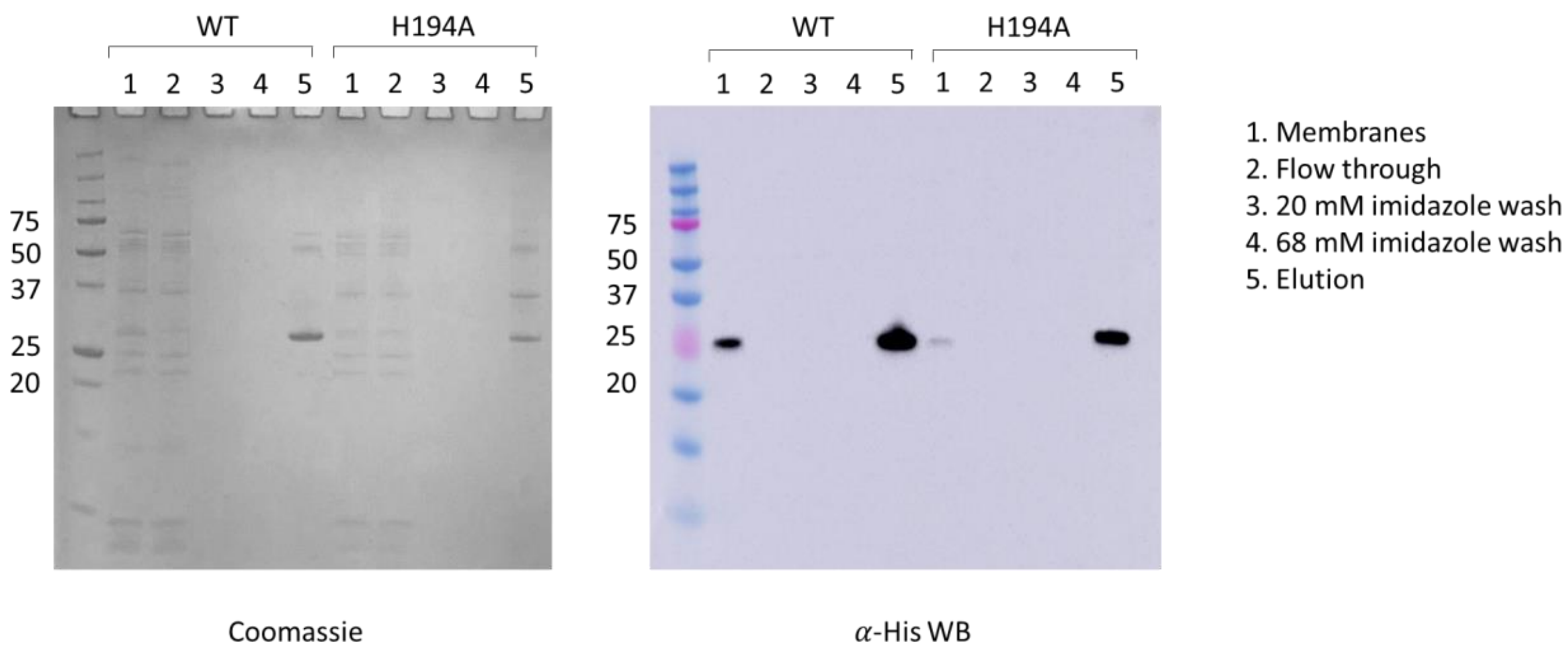

Figure S6. Western Blot analysis of strains producing wild-type (WT) and H194A variants of CrtA$^{1-241}$-His in Δ*crtA*^{stop} background. Whole cell extracts of Δ*crtA*^{stop} containing pBBRBB-Ppuc-CrtA$^{1-241}$-His with or without the H194A point mutation analysed by SDS-PAGE and immunoblotting. The gel was stained with Coomassie brilliant blue (left) and an identical gel was transferred to a membrane for immunodetection of the His-tag (right). Numbers to the left of the gel/blot indicate molecular weight in kDa and the expected molecular weight for the CrtA$^{1-241}$-His is 27 kDa.

A

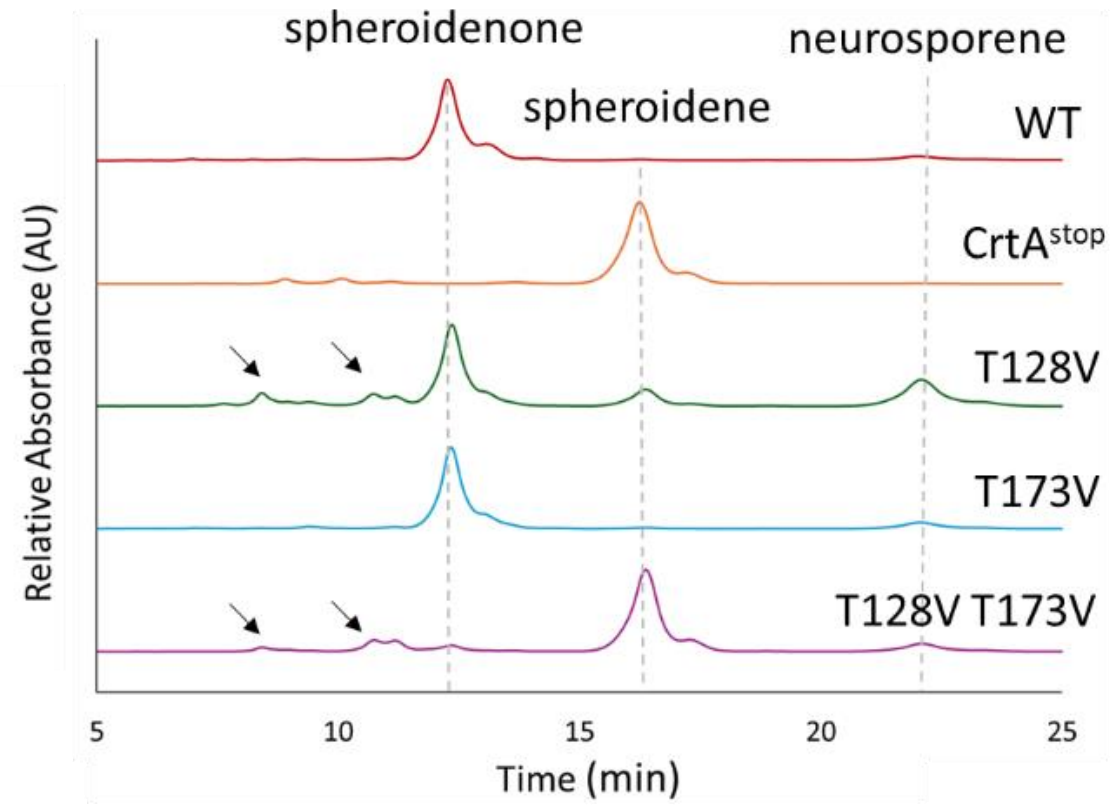

	spheroiden-one (%)	spheroid-ene (%)	neurospor-ene (%)	other
WT	98.3	0	1.7	0
CrtA^stop	0	100	0	0
T128V	55.5	9.9	21.8	12.8
T173V	95.9	0	4.1	0
T128V T173V	2.1	73.3	5.3	19.3

B

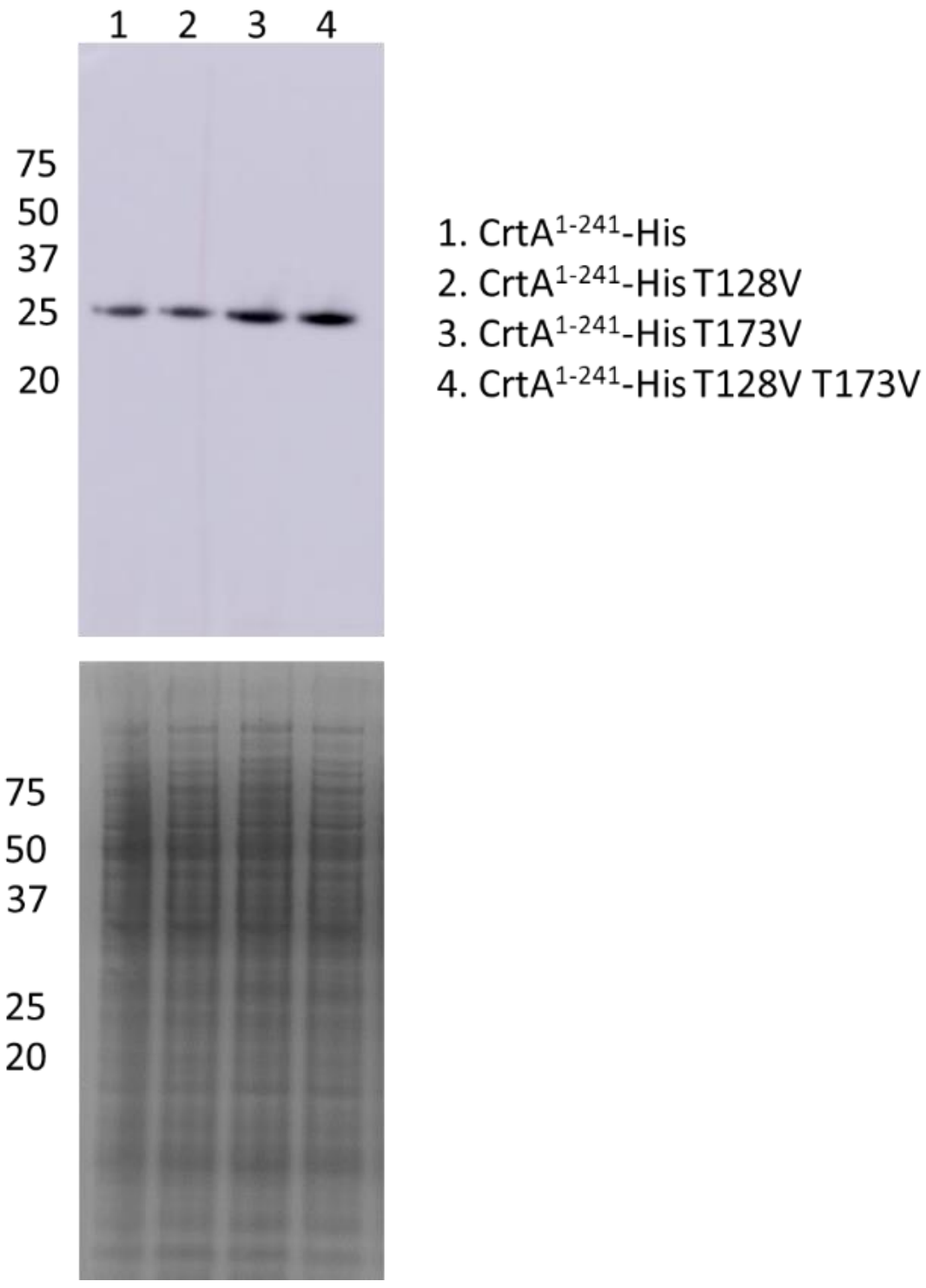

Figure S7. Western Blot analysis of point mutations of CrtA$^{1\text{-}241}$-His pBBRBB in Δ*crtA*^{stop}. (A) HPLC traces with absorption detection at 475 nm to identify the carotenoids in the strains shown in **Figure 4D**. Traces are normalised to the major peak height. Peaks assigned to known carotenoids are labelled, and those that are unassigned are identified with arrows. Relative carotenoid content is shown as a percentage, which was calculated as described in the legend of Figure S1. (B) Whole cell extracts of Δ*crtA*^{stop} containing pBBRBB-Ppuc-CrtA$^{1\text{-}241}$-His with or without various mutations of threonine to valine were analysed by SDS-PAGE normalised to cell density at 680 nm. The gel was stained with Coomassie (below) and an identical gel was transferred to membranes for immunodetection of the His-tag (above).

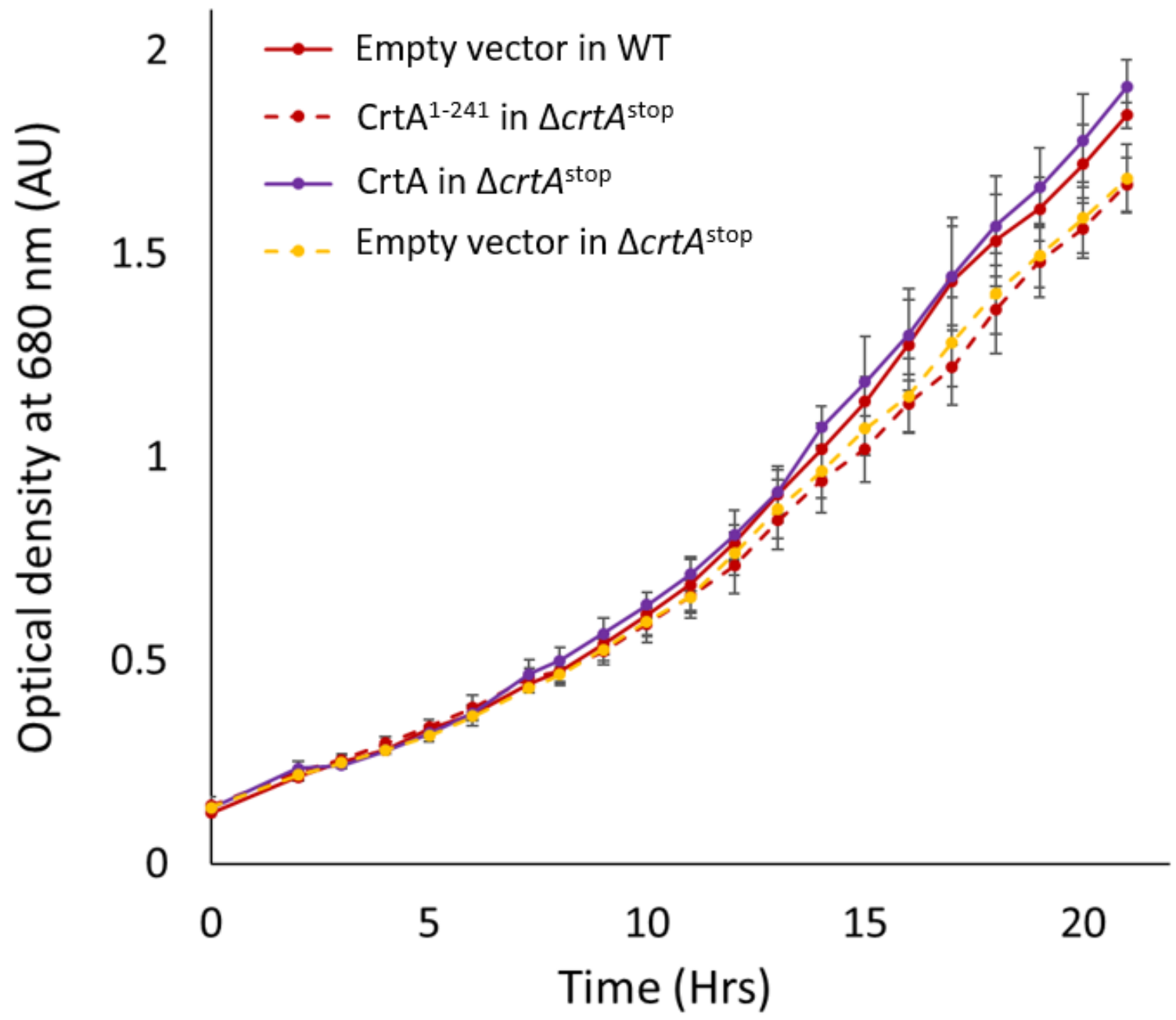

Figure S8. Growth curve comparing Δ*crtA*^{stop} complemented with pBBRBB-pPuc containing full length CrtA or CrtA$^{1-241}$ in ~30 µmol photons m^{-2} s^{-1} light conditions. Control strains expressing an empty version of the same plasmid are included for comparison. Statistical significance of the difference in growth rates was calculated by comparing growth after 21 hrs to WT. The growth rate was found to be statistically significantly different in Δ*crtA*^{stop} complemented with empty vector ($P \leq 0.005$) or CrtA$^{1-241}$ ($P \leq 0.001$) but was not when complemented with full length CrtA ($P \leq 0.065$).

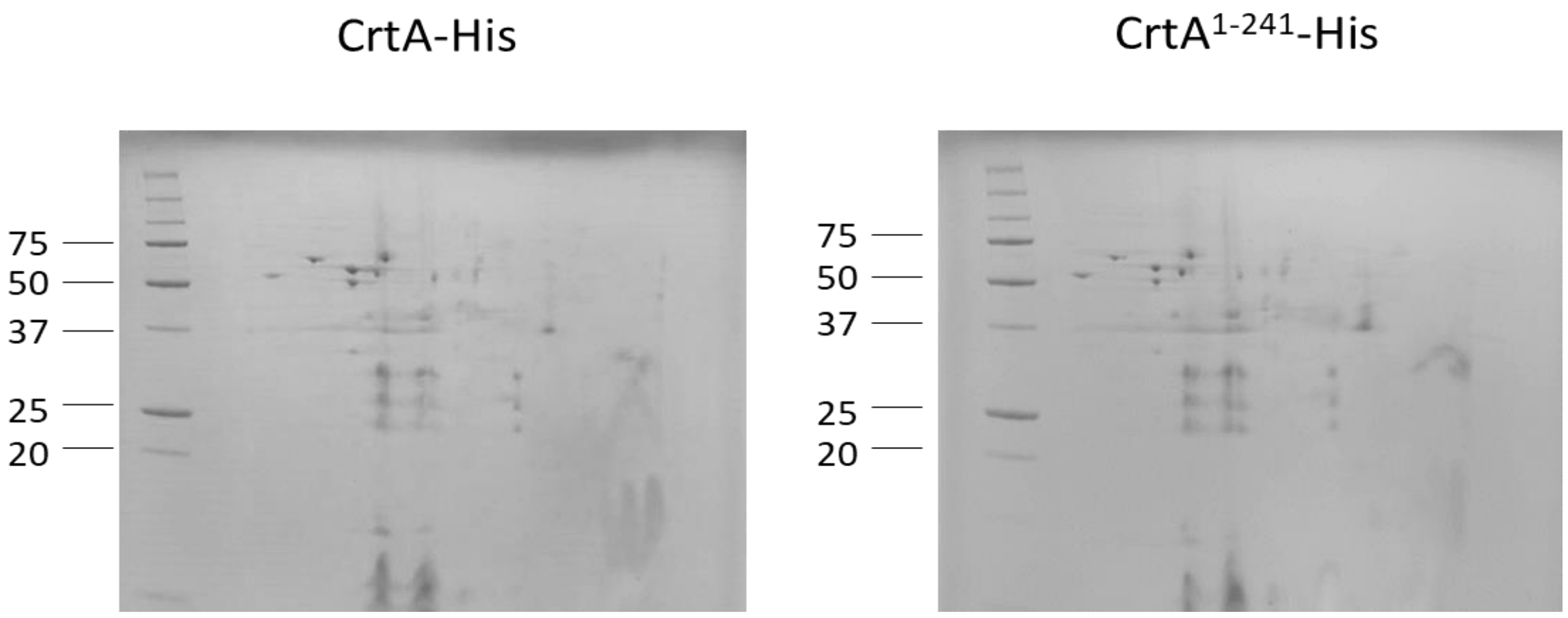

Figure S9. Coomassie stained blots of identical gels to blots shown in Figure 6C and D. Identical lanes were run on 4-16% CN-page gels, excised from the gel and analysed by SDS_PAGE in the second dimension before staining with Coomassie brilliant blue. Numbers to the left of each gel indicate molecular weight in kDa.

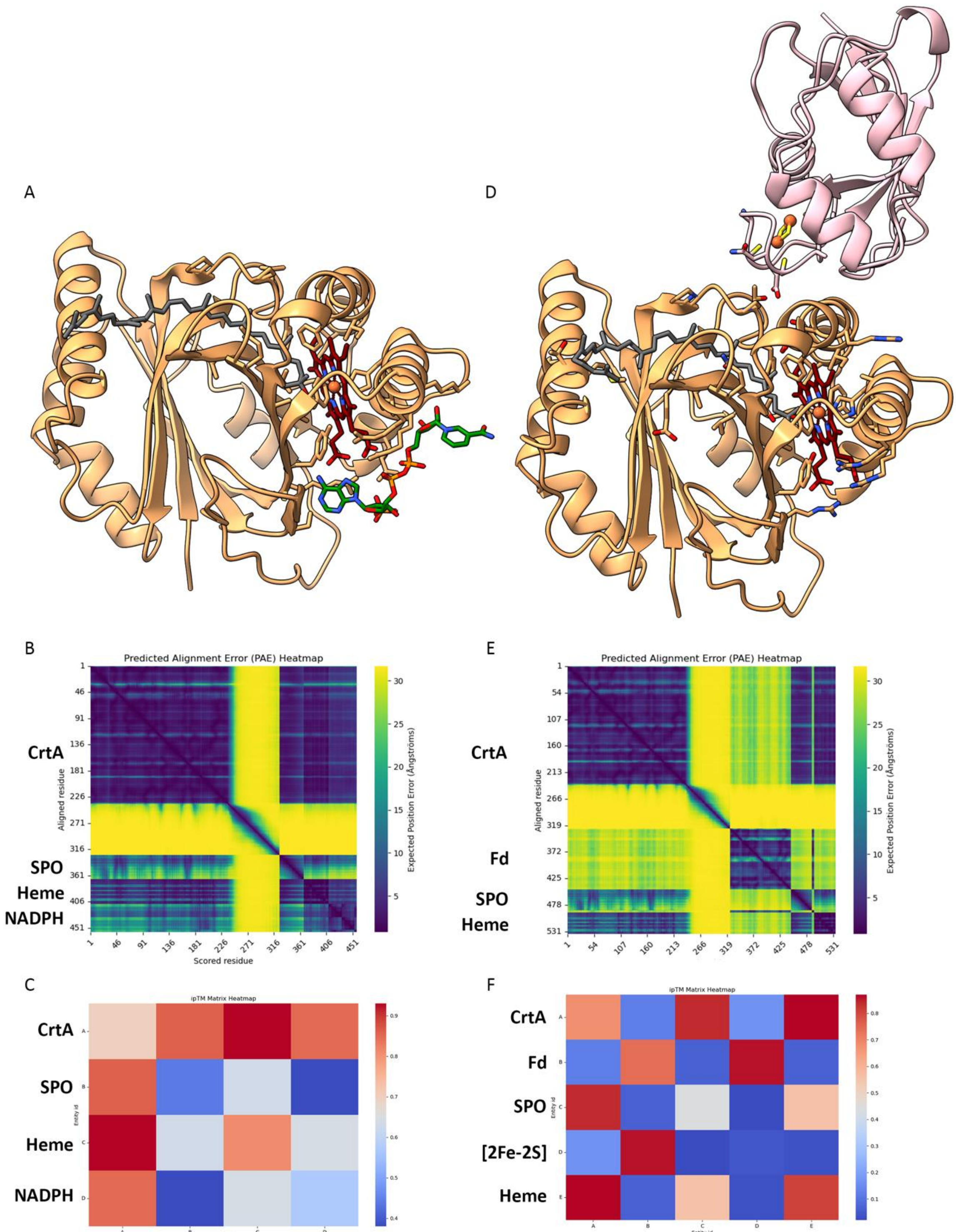

Figure S10. AlphaFold 3 modelling of CrtA with potential reducing agents for monooxygenase activity. (A) AlphaFold 3 model of CrtA (sandy brown) with NADPH (green). NADPH is predicted to dock on to the surface of CrtA near the active site and interact via S101, G195 and R214, with its redox active C4 carbon 14.9 Å away from the central heme (maroon) iron (orange)-Fe. Spheroidene is in grey. (B) Predicted Alignment Error (PAE) between CrtA, spheroidene (SPO), heme and NADPH shows low alignment error for all components except for the disordered C-terminus of CrtA. (C) Interface predicted template modelling (iPTM) shows high scores for CrtA with SPO, heme and NADPH but not

for the ligands and substrates with each other, as expected. Pertinently, the NADPH-CrtA iPTM = 0.93, predciting interaction with high confidence. (D) AF3 model of CrtA (orange) with ferredoxin (RSP_1751) (pink). The distance between the Fe atom (orange) of the heme ligand (maroon) and the 2Fe-2S cluster (orange (iron) and yellow (sulfur)) of ferredoxin is 19.2 Å. (E) PAE between components of the prediction shows low alignment error for all components except for the disordered C-terminus of CrtA and Fd, thus Fd is not predicted to bind to CrtA with high confidence. (F) iPTM for the CrtA-Fd interaction = 0.12, suggesting they do not interact.
